# The impact of skin tone on performance of pulse oximeters used by NHS England COVID Oximetry @home scheme: measurement and diagnostic accuracy study

**DOI:** 10.1136/bmj-2025-085535

**Published:** 2026-01-14

**Authors:** Daniel S Martin, James C Doidge, Doug Gould, Tasnin Shahid, Alex Cowden, Walton N Charles, Amelia Francis Johnson, Roger Garrett, Catherine Mbema, Olusegun Olusanya, Eugene Healy, Kathryn Rowan, Paul Mouncey, David A Harrison, EXAKT Study Investigators, Katie Sweet, Daniel Hill, Linda Pipira, Oluwatosin Komolafe, Marcela P Vizcaychipi, Sanjeet Chana, Laura Martins, Ilhan Wardhere, Dhinesh Sundaran, Laszlo Hollos, Anna Williams, David Golden, Rebecca Seaman, Miriam Davey, Laura Kent, Nazrudeen Ali, Ibrahim Nasr, Dee Durrant, Bincy Kariyadil, Brendan Sloan, Sarah Buckley, Amy Major, Mathew Smith, Dumindu Karangoda, Estefania Treus, Georgina Luck, Shilpa Gurung, Mandeep K Phull, Nauman Hussain, Tatiana Pogreban, Aparna George, Nicholas Truman, Qasim T Ahmed, Victoria Cunliffe, Aayesha Kazi, Mark De Neef, Poh Choo Teoh, Glykeria Pakou, Amitaa Maharajh, Sarah Penkett, Muthukumaran Gourishankar, Paul Gill, Roxanne Gray, Thomas Billyard, Carl Hawkins, Brendan B Spooner, Geraldine Ward, Monica Popescu, Theodora Christodoulopoulou, Amrinder Sayan, Jamie Gonzales, Redmond P Tully, Joy Dearden, Michelle Mulcahy, Carrole Blessen, Andrew Achilleos, Rosaleeta Reece-Anthony, Christine B Manalo, Kay Ellen Spikes, Ahilanandan Dushianthan, Michael Carter, Karen Salmon, Rachel Burnish, Christopher Hebbes, Joanna Shak, Megha Mathews, Navneet Ghuman, Alex Mattin, Geraldine Hambrook, Tom O Lawton, Omar Jundi, Sharwend Supermanian, Louise T Akeroyd, Thaventhran Prabhahar, Dhanalakshmi Bakthavatsalam, Suhieb Alhourani, Leena John, Mike Dean, Peter Mathews, Tabassum Khan, Lakshmi Aneesh, Dagan Lonsdale, Susannah Leaver, Sarah Farnell-Ward, Deborah Dawson, Nikki Yun, Maria Thanasi, Shreeja Dangol, Massimiliano Valcher, Angelo Rocha, Milafe Nimer, Frenita Dsouza, Rebecca Kanu, Maria Maiz Cordoba, Vincent Ventura, Edna Fernandes, Karen Lloyd, John Adam, Rohit Saha, Tom Williams, Kevin O'Reilly, Anna Broderick

**Affiliations:** 1 Peninsula Medical School, University of Plymouth, Plymouth, UK; 2 Intensive Care Unit, University Hospitals Plymouth NHS Trust, Plymouth, UK; 3 Clinical Trials Unit, Intensive Care National Audit & Research Centre (ICNARC), London, UK; 4 Independent patient advisor, Bristol, UK; 5 Lewisham Council, London, UK; 6 Department of Perioperative Medicine, St Bartholomew’s Hospital, London, UK; 7 Dermatopharmacology, University of Southampton, Southampton General Hospital, Southampton, UK; 8 Dermatology, University Hospital Southampton NHS Foundation Trust, Southampton, UK

## Abstract

**Objectives:**

To assess the impact of skin tone on the measurement and diagnostic accuracy of five fingertip pulse oximeters used by patients in the NHS (National Health Service) England COVID Oximetry @home scheme.

**Design:**

Measurement and diagnostic accuracy study (exploring pulse oximeter accuracy across skin tones—EXAKT).

**Setting:**

Twenty four intensive care units in England between June 2022 and August 2024.

**Participants:**

903 critically ill adults admitted to intensive care units screened for or enrolled into a trial evaluating different approaches to oxygen therapy.

**Interventions:**

Pulse oximetry derived peripheral oxygen saturation (SpO_2_) measurements were compared with paired arterial oxygen saturation (SaO_2_) measurements from arterial blood analysed by co-oximetry (gold standard). Skin tone (individual typology angle) was objectively measured using a handheld spectrophotometer.

**Main outcome measures:**

Pulse oximeter measurement accuracy was assessed for bias, precision, and overall accuracy. Diagnostic accuracy for identifying SaO_2_ ≤92% was assessed by false negative and false positive rates for SpO_2_ using thresholds of ≤92% and ≤94%, and the area under the receiver operating characteristic curve, and by the presence of occult hypoxaemia (SaO_2_ <88% with SpO_2_ >92%).

**Results:**

11 018 paired SpO_2_-SaO_2_ measurements were analysed. All tested pulse oximeters overestimated at lower values and underestimated at higher values of SaO_2_. On average, SpO_2_ readings were 0.6-1.5 percentage points higher for patients with darker skin tone (individual typology angle −44°) than for those with lighter skin tone (46°). At both SpO_2_ thresholds assessed, false negative rates increased with darker skin tones; the proportion of SpO_2_ measurements >94% despite a paired SaO_2_ ≤92% ranged from 5.3 to 35.3 percentage points higher for patients with darker skin tones than for those with lighter skin tones (7.6-62.2% *v* 1.2-26.9%, rate ratio 2.3-7.1). By contrast, false positive rates decreased with darker skin tones.

**Conclusions:**

Five pulse oximeters provided by the NHS England COVID Oximetry @home scheme yielded higher SpO_2_ measurements for patients with darker skin tones compared with those with lighter skin tones, which could translate into potentially clinically important differences in false negative and false positive rates for detecting hypoxaemia.

**Trial registration:**

ClinicalTrials.gov NCT05481515

## Introduction

Arterial haemoglobin oxygen saturation (SaO_2_) indicates the fraction of oxygenated haemoglobin, relative to total haemoglobin, in arterial blood and provides a useful indicator of hypoxaemia (low blood oxygen levels). The gold standard measurement for SaO_2_ is an arterial blood sample analysed by the co-oximeter of a blood gas machine. Pulse oximetry is a technology developed to estimate SaO_2_ non-invasively by measuring the absorbance of two wavelengths of light (660 and 940 nm) transmitted across the vascular bed of a tissue. Differential light absorbance characteristics between oxygenated and deoxygenated haemoglobin permits the calculation of a ratio, allowing estimation of SaO_2_. Pulse oximetry is widely used throughout the world to monitor patients in healthcare settings and, more recently, in the community.

Since pulse oximeters first became commercially available in the 1980s, advances in their design have led to the development of low cost, battery powered, portable devices that can be used at home by the public. During the covid-19 pandemic, these fingertip pulse oximeters were supplied to people at home to enable remote monitoring of peripheral oxygen saturation (SpO_2_) and support early detection of hypoxaemia.^1^ In England, the National Health Service (NHS) introduced the COVID Oximetry @home scheme. The effectiveness of such schemes has been difficult to evaluate retrospectively owing to the complexity of the pandemic and several confounding factors.^2^ ^3^ ^4^ Some low cost pulse oximeters have been shown to be inaccurate, with their accuracy falling short of that required for regulatory approval.^5^ Studies have also shown that SaO_2_ is overestimated by pulse oximetry in people with darker skin tones.^6^ Overestimation of SaO_2_ by pulse oximetry could lead to the late or absent diagnosis of hypoxaemia and subsequent delay or failure to start life-saving treatments.^7^ The magnitude of any overestimation has been difficult to quantify because of the limitations of studies published to date. Many studies have used ethnicity or race as a proxy for skin tone, while others have assessed skin tone using the Fitzpatrick skin type or other colour comparison charts. Both methodological approaches have major limitations that can be avoided by obtaining objective measurements of skin tone using spectrophotometry.^8^ ^9^

The EXAKT (exploring pulse oximeter accuracy across skin tones) study was commissioned and rapidly funded by the National Institute for Health and Care Research in response to concerns raised by the NHS in England about inaccuracies in pulse oximeters (HTA 21/608, 2022). The aim was to assess the measurement and diagnostic accuracy of five fingertip pulse oximeters provided by the NHS for use at home in the NHS England COVID Oximetry @home scheme and to assess the impact of skin tone on accuracy.

## Methods

### Sites and participants

The EXAKT study took advantage of the screening infrastructure for an ongoing, large multicentre randomised clinical trial, the UK-ROX trial.^10^ Participants were adult patients (18 years and older) receiving mechanical ventilation and supplementary oxygen in 24 NHS adult intensive care units in England. Patients were recruited to the EXAKT study from those enrolled into—and those screened but not enrolled into—the UK-ROX trial. Selection was weighted to ensure over-representation of patients with darker skin tones (see supplementary methods). Ethical approval was granted by the South Central Oxford C Research Ethics Committee (20/SC/0423) and the EXAKT protocol was prospectively registered with ClinicalTrials.gov (NCT05481515). The supplementary methods provide full details of eligibility criteria and consent procedures. We linked patient data recorded for the EXAKT study with routinely collected data from the ICNARC Case Mix Programme, the national clinical audit of adult critical care, to provide information on patient characteristics. No aspect of patients’ clinical care was altered as a result of this study. Readings from the study pulse oximeters being evaluated were not used when making clinical decisions. A separate pulse oximeter linked to a critical care monitor remained attached to each patient throughout the study for clinical decision making. The critical care pulse oximeters were not evaluated for accuracy in this study.

### Pulse oximeters

We evaluated five battery powered, fingertip pulse oximeters provided by NHS England from those in use in the COVID Oximetry @home scheme^4^: ChoiceMMed Oxywatch MD300C19 (FBC3776), ChoiceMMed Oxywatch MD300C13-CC12 (FBC3826), Creative Medical PC-60B1-BL (FBC3828), Biolight Meditech M70C (FBC3831), and Medlinket AM802 NHE (FBC3830). Two different pulse oximeter models were randomly allocated to each participating site. As the EXAKT study progressed, pulse oximeter use was regularly reviewed and oximeters moved around participating sites to ensure sufficient recruitment for each oximeter.

### Measurements of arterial oxygenation

The index test measure was pulse oximetry derived SpO_2_ and the reference standard was functional SaO_2_ measured by the co-oximeter of a standard blood gas machine (gold standard). Contemporaneous, paired SpO_2_-SaO_2_ measurements were collected over a 24 hour period for each patient. Immediately before routine arterial blood gas sampling from an indwelling arterial catheter, each of the two study pulse oximeters were placed on fingers of the same hand of the patient by a member of the clinical team. The supplementary methods provide details of placement of pulse oximeters. After a period of stabilisation, the SpO_2_ value from each device was recorded and the arterial blood gas sample was immediately taken. SaO_2_ values from the co-oximeter were not corrected for patient temperature. A list of blood gas machines used in the study is provided in the supplementary methods.

### Measurement of skin tone

Skin tone was measured for each patient using a handheld spectrophotometer (Konica Minolta CM-600d; see supplementary methods). In brief, four measurements were taken from normal skin on the dorsal surface of each patient’s non-dominant hand, making sure to avoid any pigmented lesions on the skin. The International Commission on Illumination L*, a*, and b* values for each reading were recorded. L* represents dark through to light (correlating with skin colour); a* represents cutaneous erythema and is affected by melanin composition and cutaneous blood flow; and b* reflects a person’s constitutional pigmentation and their ability to tan.^11^ The median L* and b* value for each patient were used to calculate the individual typology angle (ITA) as an objective continuous measure of skin tone.^12^ We used previously defined categories of skin tone based on ITA in descriptive results (very light: ITA≥55°; light: 41°≤ITA<55°; intermediate: 28°≤ITA<41°; tan: 10°≤ITA<28°; brown: −30°≤ITA<10°; dark: ITA less than −30°).^12^ ^13^ For simplicity, when describing results, some comparisons are made between patients with median dark (the observed median ITA among patients with ITA less than −30°) and median very light or light skin tone (median among patients with ITA ≥41°).

### Assessment of accuracy of pulse oximeters

Measurement accuracy was assessed by bias (systematic error), precision (random error or noise), and their combination for overall accuracy (assessed by accuracy root mean square, A_RMS_; see supplementary methods for definitions). Diagnostic accuracy was assessed by false negative rate (one minus sensitivity) and false positive rate (one minus specificity) of SpO_2_ ≤92% and SpO_2_ ≤94% for detecting SaO_2_ ≤92%, and their combination as the area under the receiver operating characteristic curve (AUC) across all possible thresholds of SpO_2_ for detecting SaO_2_ ≤92%. SpO_2_ thresholds were selected to correspond with the COVID Oximetry @home guidance to seek medical help (≤94%) or to attend the emergency department (≤92%).^14^ Occult hypoxaemia was assessed as the proportion of patients with SaO_2_ <88% who had SpO_2_ >92%. The impact of skin tone (ITA) on performance was assessed for all seven metrics.

### Statistical analysis

Simulations informed by data from the UK-ROX trial and Case Mix Programme indicated that a sample of 900 patients yielding an anticipated 10 800 paired SpO_2_-SaO_2_ measurements would give at least 90% power to detect a linear variation in bias by skin tone corresponding to a 1.5 percentage point difference in bias between ITA values of 28° (lower boundary of the intermediate category) and −30° (upper boundary of the dark category).

For each accuracy metric, a single fractional probit regression model was fitted to the set of complete observations, adjusted for fixed effects of pulse oximeter model, ITA, SaO_2_, and haemoglobin concentration, and interactions between pulse oximeter model and ITA, pulse oximeter model and SaO_2_, and ITA and SaO_2_. ITA, SaO_2_, and haemoglobin concentration were modelled continuously using restricted cubic splines. To assess bias, this model was fitted with SpO_2_ as the dependent variable. To assess precision, this model was fitted with the squared deviation between SpO_2_ and the marginal predicted SpO_2_ from the bias model as the dependent variable. To assess accuracy, this model was fitted with the squared difference between SpO_2_ and paired SaO_2_ as the dependent variable to estimate A_RMS_. Models included fixed effects of site and standard errors that account for repeated measures within patients.

To estimate false negative and false positive rates, multilevel logistic regression models were fitted with binary dependent variables of SpO_2_ ≤92% and SpO_2_ ≤94%. Except for the addition of random effects for patients within sites, these models had identical structure to the models above. AUC was estimated using a probit receiver operating characteristic regression model with a dependent variable of SaO_2_ ≤92%, independent variable of SpO_2_ and adjusted for fixed effects of pulse oximeter model, ITA, and interactions between pulse oximeter model and ITA. The rate of occult hypoxaemia was estimated as the marginal predicted probability of SpO_2_ >92% averaged over the distribution of SaO_2_ among patients with SaO_2_ <88%.

The supplementary methods provide full details of statistical analyses and regression model outputs. The full analysis code can be viewed at https://github.com/ICNARC/EXAKT. Analyses were conducted using Stata/MP version 18.0.

### Patient and public involvement

A patient advisor with lived experience was involved with the design of the study, the funding application (as a named applicant), the conduct of the study (as a member of the trial management group), and the editing of this manuscript (as a named author, RG). The study was designed following the National Institute for Health and Care Research INCLUDE Ethnicity Framework.^15^ Given the nature of the research, we involved a public health professional (CM) with experience leading community involvement work with black, Asian, and other minoritised communities during the covid-19 pandemic. She helped our research team to form a community engagement partnership with Liberating Knowledge, who share experiences of belonging to groups racialised as minorities. Liberating Knowledge has enabled us to reach and communicate with communities who are likely to be most affected by the results of this study. We have worked with them to understand the current issues surrounding the area we studied and how to disseminate these new findings in a way that they are meaningful to those they may affect. Specifically, through Liberating Knowledge, we identified two public representatives to join our study management group meetings and provided input into the oversight of the study conduct. We hosted co-design workshops with members of voluntary, community, and social enterprise organisations, research teams recruiting into the EXAKT study and members of the community. These workshops aimed to explore the challenges faced in recruiting people from black and South Asian communities and explore opportunities to raise awareness and identify approaches to increase the engagement of the wider community and identify the best ways to disseminate the results of the study.

In addition to these activities, we produced regular blog posts focused on the study and our engagement activities, and presented at Health and Racial Justice Laboratories to raise awareness of the study more widely. Further to our work with Liberating Knowledge, during the design phase of the study, we worked alongside the Centre for Ethnic Health Research, and hosted focus groups with public representatives to ensure we approached potential participants and their families appropriately and to review the information provided to patients recruited into EXAKT. Because our study focused on patients with darker skin tones, we needed to understand how the patients and their relatives would feel about being approached to participate in a study related to skin tone.

## Results

### Participant characteristics

Between June 2022 and August 2024, 903 patients were recruited from 24 sites and included in the analysis (supplementary figure 1; one patient was omitted from statistical models because of missing haemoglobin in all observations). Table 1 and supplementary table 1 summarise patient characteristics. Of the 903 patients, 848 had linked routinely collected data available (94%; 566 male, 282 female; mean age 56 years); 232 (31%) were from a white ethnic group (lower than the UK population because of weighted sampling), 227 (31%) were Asian, 177 (24%) were black, and 101 (14%) were from a mixed or other ethnic group (ethnic group not available or not stated for 111 patients).

**Table 1:** Patient characteristics and arterial blood gas observations

Patient characteristics and arterial blood gas observations	Pulse oximeter					Overall
	ChoiceMMed Oxywatch MD300C19	ChoiceMMed Oxywatch MD300C13-CC12	Creative Medical PC-60B1-BL	Biolight Meditech M70C	Medlinket AM802 NHE	
No of patients	369	351	351	346	388	903
Age,* mean (SD)	56.1 (16.1); 348	55.4 (15.8); 329	55.7 (15.4); 337	55.6 (16.2); 312	55.0 (16.8); 369	55.6 (16.0); 848
Male*	234 (67.2); 348	217 (66.0); 329	234 (69.4); 337	193 (61.9); 312	253 (68.6); 369	566 (66.7); 848
Ethnic group†						
White	104 (33.3); 312	99 (35.7); 277	75 (25.3); 296	88 (32.1); 274	98 (31.2); 314	232 (31.5); 737
Asian	104 (33.3); 312	83 (30.0); 277	111 (37.5); 296	66 (24.1); 274	90 (28.7); 314	227 (30.8); 737
Black	72 (23.1); 312	52 (18.8); 277	72 (24.3); 296	84 (30.7); 274	73 (23.2); 314	177 (24.0); 737
Mixed or other	32 (10.3); 312	43 (15.5); 277	38 (12.8); 296	36 (13.1); 274	53 (16.9); 314	101 (13.7); 737
Skin tone group						
Very light or light‡	46 (12.5)	54 (15.4)	29 (8.3)	46 (13.3)	47 (12.1)	111 (12.3)
Intermediate	60 (16.3)	53 (15.1)	57 (16.2)	47 (13.6)	61 (15.7)	139 (15.4)
Tan	77 (20.9)	79 (22.5)	78 (22.2)	74 (21.4)	88 (22.7)	198 (21.9)
Brown	122 (33.1)	115 (32.8)	112 (31.9)	124 (35.8)	137 (35.3)	305 (33.8)
Dark	64 (17.3)	50 (14.2)	75 (21.4)	55 (15.9)	55 (14.2)	150 (16.6)
Chronic respiratory disease§	24 (7.6); 317	28 (9.3); 300	13 (4.9); 267	18 (6.9); 259	15 (4.4); 344	49 (6.6); 744
Reason for admission to critical care (body system)*						
Respiratory	105 (30.2); 348	101 (30.7); 329	100 (29.7); 337	95 (30.4); 312	99 (26.8); 369	250 (29.5); 828
Cardiovascular	70 (20.1); 348	57 (17.3); 329	59 (17.5); 337	70 (22.4); 312	82 (22.2); 369	169 (19.9); 828
No of arterial blood gas observations	2177	2215	2010	2355	2251	5656
SaO_2_ (%), median (IQR)	96 (94-98)	95 (93-97)	96 (94-98)	96 (94-97)	96 (94-98)	96 (94-97)
SaO_2_ (%) range¶						
<88	30 (1.4)	36 (1.6)	28 (1.4)	35 (1.5)	44 (2.0)	95 (1.7)
88-92	306 (14.1)	391 (17.7)	245 (12.2)	322 (13.7)	284 (12.6)	796 (14.1)
93-94	357 (16.4)	414 (18.7)	322 (16.0)	400 (17.0)	339 (15.1)	940 (16.6)
>94	1484 (68.2)	1374 (62.0)	1415 (70.4)	1598 (67.9)	1584 (70.4)	3825 (67.6)
Haemoglobin (g/L), median (IQR)	101 (86-116)	104 (90-123)	99 (85-113)	100 (86-117)	99 (85-115)	101 (86-117)
Carboxyhaemoglobin** (%), median (IQR)	1.1 (0.7-1.5)	1.2 (0.8-1.5)	1.2 (0.8-1.5)	1.2 (0.8-1.5)	1.2 (0.9-1.5)	1.2 (0.8-1.5)
Methaemoglobin†† (%), median (IQR)	0.7 (0.5-1.0)	0.8 (0.5-1.1)	0.7 (0.3-1.0)	0.6 (0.4-0.9)	0.8 (0.5-1.1)	0.7 (0.4-1.0)

Data are presented as number (%) unless stated otherwise. Number of patients with available data is also given when not complete (number (%); No of patients).

IQR=interquartile range; SaO_2_=arterial oxygen saturation; SD=standard deviation; SpO_2_=peripheral oxygen saturation.

* Based on linked routine data; n=55 (6.1%) not linked.

† Based on linked routine data; n=55 (6.1%) not linked, n=111 (12.3%) recorded as “Not stated.”

‡ Owing to small numbers, very light (n=13) is combined with light in tables and figures.

§ Shortness of breath with light activity owing to pulmonary disease and evident within six months before admission. Based on linked routine data; n=55 (6.1%) not linked, n=104 (11.5%) missing.

¶ Rounded to nearest percentage point, for consistency with SpO_2_.

** n=36 (0.6%) missing.

†† n=71 (1.3%) missing.

### Summary of key measures

**Fig 1:**
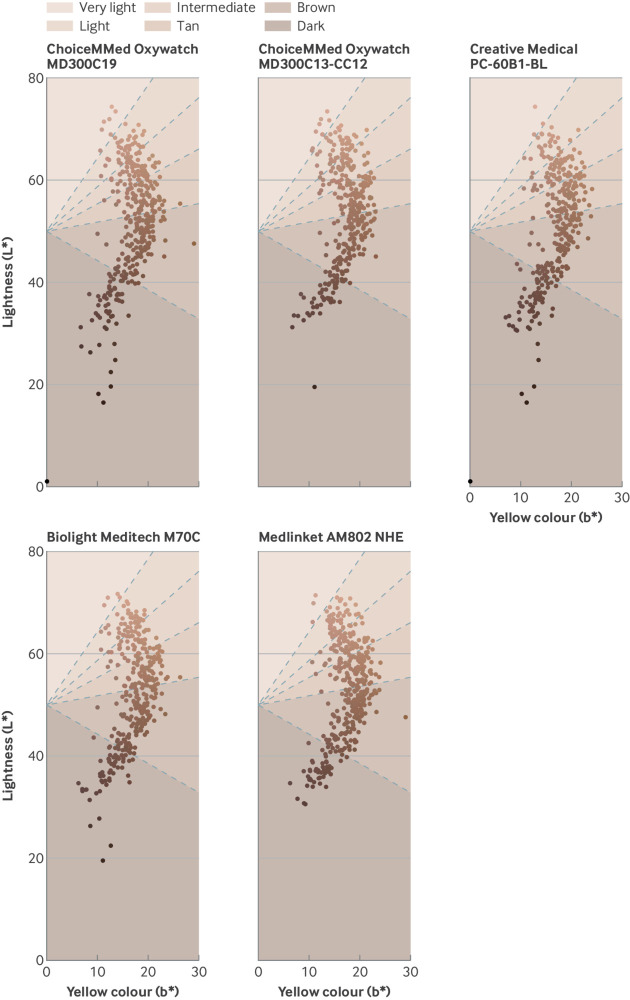
Distribution of skin tone in the EXAKT (exploring pulse oximeter accuracy across skin tones) study cohort by pulse oximeter. Each dot represents one patient in EXAKT study cohort, with each value (lightness L* and yellow components b*) averaged across four spectrophotometer readings of dorsal aspect of hand. Colours displayed are approximately indicative of patient’s skin tone and may be affected by display settings or print quality. Individual typology angle indicated by angle above or below horizontal line from L*=50, b*=0, with boundaries between skin tone categories visualised as dashed lines at 55°, 41°, 28°, 10°, and −30°

A total of 5656 measurements of SaO_2_ were collected, yielding 11 018 observation points when paired with synchronous SpO_2_ measurements from up to two pulse oximeters for each patient. Median SaO_2_ was 96% (interquartile range 94-97%) and 891 (16%) measurements were of SaO_2_ ≤92% (table 1). Figure 1 and supplementary figure 2 show the range of participant skin tones described by ITA and skin tone category. The median within-patient standard error of ITA estimation was 3°.

**Fig 2:**
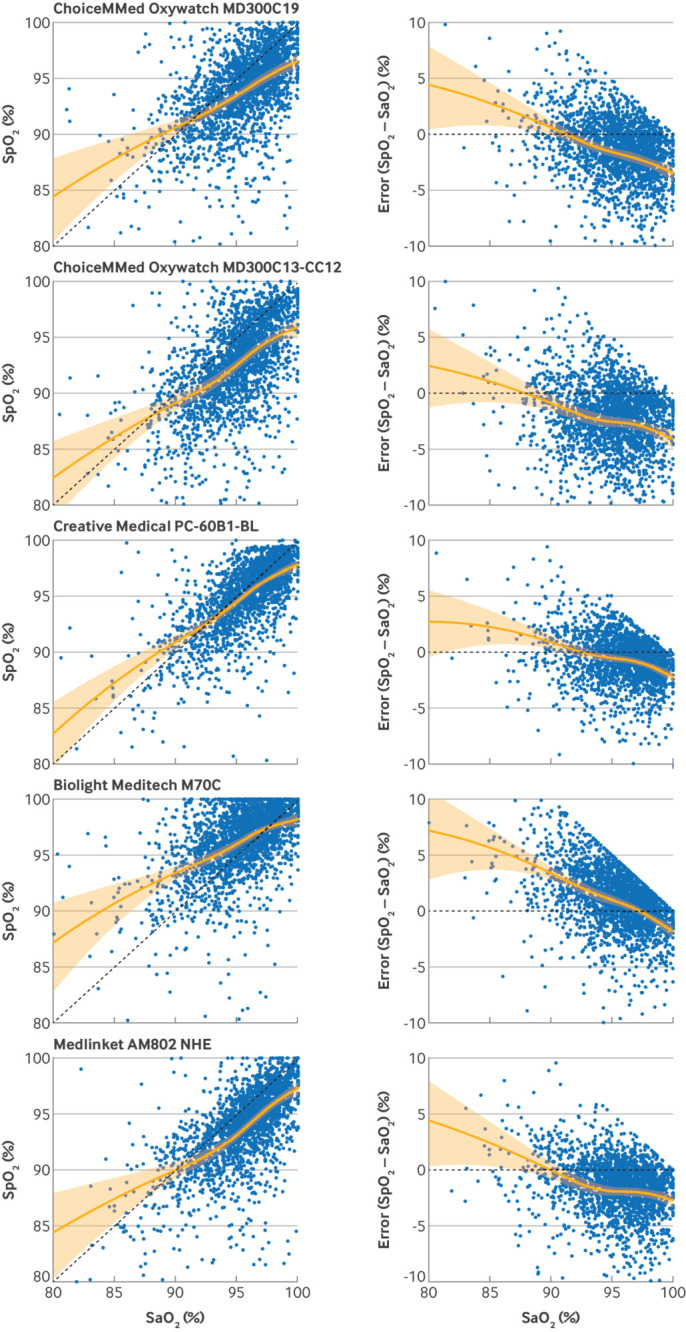
Association between SpO_2_ (peripheral oxygen saturation) and SaO_2_ (arterial oxygen saturation) by pulse oximeter. Each dot represents one observation showing association between SpO_2_ and SaO_2_ across range of SaO_2_ from 80% to 100%, with dashed line indicating exact agreement. Left panels: data are presented with SpO_2_ on vertical axis; right panels: same data are presented with difference between SpO_2_ and SaO_2_ (error or bias) on vertical axis. Solid lines show adjusted mean error (bias) with shaded 95% confidence intervals

Figure 2 and supplementary figures 3A–E show the association between SpO_2_ and SaO_2_. All pulse oximeters tended to overestimate at lower values of SaO_2_ and underestimate at higher values of SaO_2_ (as indicated by the solid orange regression lines positioned above or below the dashed green lines of exact agreement), but with substantial imprecision at all levels of SaO_2_ (as indicated by the scattered observation points). Variation existed across the pulse oximeters in the degree of overestimation and underestimation, and the tipping point at which overestimation switched to underestimation (where the regression line crosses the line of exact agreement).

### Effect of skin tone on pulse oximeter accuracy

**Fig 3:**
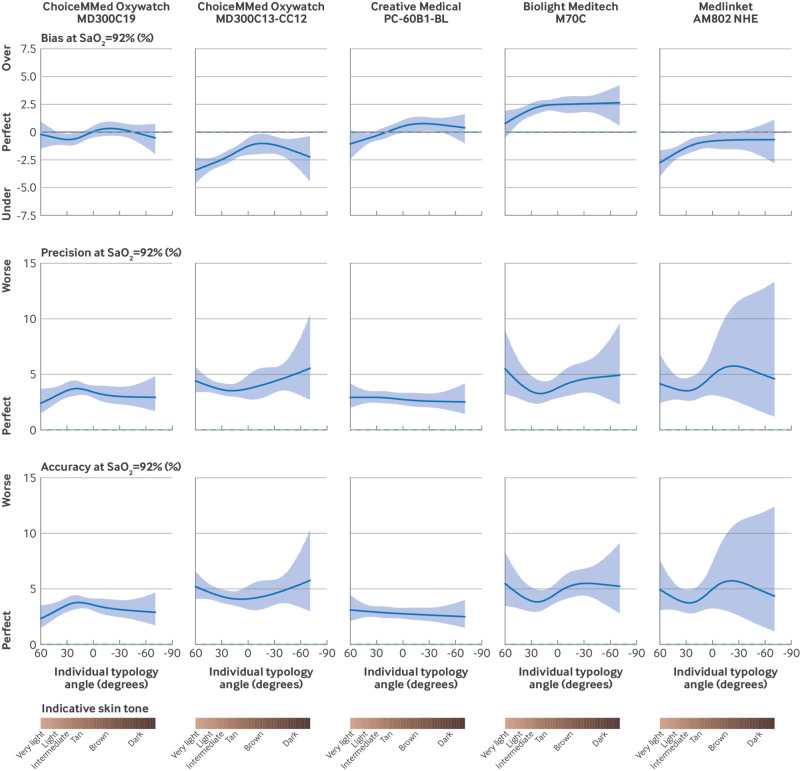
Variation in measurement accuracy at SaO_2_ (arterial oxygen saturation) 92%, across range of skin tones. Solid lines indicate modelled estimates, with shading of 95% confidence limits. Dashed lines indicate exact agreement or perfect prediction. Bias: mean error (systematic error); precision: root mean squared deviation (random error); accuracy: accuracy root mean square (total error). See supplementary methods for full definitions, details of statistical modelling, and estimates at different levels of SaO_2_

All pulse oximeters tended to give higher SpO_2_ readings at darker skin tones (fig 3, supplementary tables 2-4, and supplementary figures 4-7). For any given SaO_2_ across the range studied, SpO_2_ readings were on average 0.6-1.5 percentage points higher for a patient with median dark skin tone (ITA −44°) than for a patient with median light skin tone (ITA 46°; supplementary table 2). However, because all pulse oximeters tended to underestimate high saturations and overestimate low saturations, the higher readings for darker skin tones could translate into increasing positive bias or decreasing negative bias, depending on the SaO_2_ (worse bias for patients with low saturation but potentially less bias for patients with higher saturations; see supplementary figure 4). Consequently, there were no clear and consistent patterns in accuracy, as assessed by A_RMS_, across skin tones (supplementary figure 6).

### Effect of skin tone on false positive and false negative rates for detecting hypoxaemia

**Fig 4:**
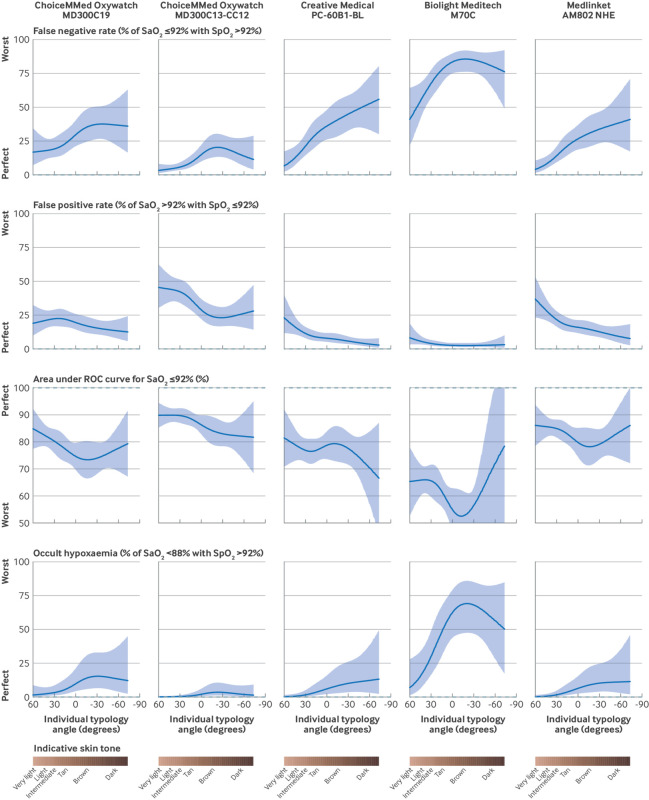
Variation in diagnostic accuracy to detect SaO_2_ (arterial oxygen saturation) ≤92% across range of skin tones. Solid lines indicate modelled estimates, with shading of 95% confidence limits. Dashed lines indicate exact agreement or perfect prediction. False negative rate: failure of pulse oximeter to correctly identify hypoxaemia; false positive rate: pulse oximeter indicating hypoxaemia when no hypoxaemia present; area under receiver operative characteristic (ROC) curve: likelihood that pulse oximeter will indicate lower oxygen saturation for a person with hypoxemia than for a person without hypoxemia; occult hypoxaemia: failure of pulse oximeter to identify severe hypoxaemia. See supplementary methods for full definitions, details of statistical modelling, and estimates at different levels of SpO_2 _(peripheral oxygen saturation)

For all pulse oximeters and at both thresholds assessed (SpO_2_ ≤92% and SpO_2_ ≤94%), the false negative rate for identifying SaO_2_ ≤92% increased with darker skin tone (fig 4, supplementary tables 5-8, and supplementary figure 8). For example, across the five pulse oximeters, the false negative rate for SpO_2_ ≤94% (ie, the proportion of SpO_2_ readings >94% despite an associated SaO_2_ ≤92%) ranged from 5.3 to 35.3 percentage points higher for a patient with median dark skin tone than for a patient with median light skin tone (7.6-62.2% *v* 1.2-26.9%, rate ratio 2.3-7.1). Conversely, the false positive rate for identifying SaO_2_ ≤92% decreased with darker skin tone. Absolute values of the false negative and false positive rates were driven by the overall bias of the pulse oximeter model, with pulse oximeters that tended to underestimate SaO_2_ having lower false negative rates and higher false positive rates, and pulse oximeters that tended to overestimate SaO_2_ having higher false negative rates and lower false positive rates. The overall discriminatory power of SpO_2,_ to predict an SaO_2_ ≤92%, assessed as the area under the receiver operative characteristic curve, tended to decrease with darker skin tone (fig 4). Estimated rates of occult hypoxaemia were imprecise owing to the small number of observations with SaO_2_ <88%, but tended to increase with darker skin tone (fig 4). The supplementary methods show details of regression model outputs.

## Discussion

### Principal findings

We found that all five fingertip pulse oximeters supplied for home use in the NHS England COVID Oximetry @home scheme returned higher SpO_2_ values for patients with darker skin tones than patients with lighter skin tones at any given level of SaO_2_ measurement. The nature and magnitude of this bias varied between pulse oximeters and depended on an individual’s skin tone and the underlying SaO_2_. At high saturations, all pulse oximeters underestimate SaO_2_ and higher readings for darker skin tones counterintuitively mean less bias. However, each pulse oximeter showed a tipping point below which SaO_2_ was overestimated and darker skin tone increased the bias.

Overall measurement accuracy (A_RMS_) was primarily driven by precision and was not sensitive to the relatively small variations in bias by skin tone. However, these small variations in bias led to marked differences in diagnostic accuracy, with much higher false negative rates (failure to identify true hypoxaemia, or occult hypoxaemia) but correspondingly lower false positive rates (false alarms) in patients with darker skin tones. Although the associations between skin tone and accuracy were non-linear, the clinically important associations with bias, false negative rates, and false positive rates were generally monotonic (always increasing or decreasing with skin tone, within the bounds of the confidence intervals shown).

### Comparison with other studies

Our findings align with laboratory investigations of healthy volunteers, smaller clinical studies, and retrospective database analyses conducted over the past four decades that have reported associations between skin tone and diagnostic accuracy.^6^ However, our findings undermine the simplistic view that oximeter function is worse in patients with darker skin tones. Of note, two of the largest prospective clinical accuracy studies to date reported conflicted findings. A study of 394 critically ill patients in intensive care units reported that bias was greater in patients with darker rather than lighter skin tones,^16^ while the other study recruited 400 hospital inpatients and outpatients but found that skin tone did not affect pulse oximeter accuracy.^17^ Both studies differed from ours in that they used subjective measures of skin tone.

The degree to which pulse oximeters overestimate SaO_2_ in people with darker skin tones has been difficult to quantify because it varies between devices, is exacerbated by low SaO_2_, and many studies have used ethnicity as a proxy measure for skin tone. Our study evaluated five low cost pulse oximeters, but did not compare measurements with those from devices commonly used in a hospital setting. The devices were supplied to the study team through the procurement route used to provide oximeters for the NHS England COVID Oximetry @home scheme and represent the devices used by the programme at that time. Other similar devices are available and may be in use both within the scheme and elsewhere. The findings of this study may not be generalisable to other models of pulse oximeters in different clinical settings. However, in a recent, much smaller study using a similar design to ours, investigators reported the accuracy of a United States Food and Drug Administration approved critical care pulse oximeter using data from 350 paired SpO_2_-SaO_2_ measurements from 12 critically ill adults, five with dark and seven with lighter skin tone.^18^ Using the same spectrophotometer as used in our study to calculate ITA, the investigators reported considerable overestimation of SaO_2_ by the pulse oximeter for patients with darker skin tones. In another study, spectrophotometry was used in a laboratory setting to measure the ITA of healthy volunteers exposed to hypoxic gas mixtures while evaluating the accuracy of 11 different pulse oximeters.^8^ The investigators reported that five of the 11 devices had an A_RMS_ >3% and that nine performed worse for participants with the darkest skin tones. Comparison of our study to most of the other published literature in this field is difficult because other studies did not measure skin tone directly, or did so using colour charts, a method know to be flawed.^19^ ^20^

### Strengths and limitations of this study

The strengths of our study include the large number of participants and simultaneous prospective measurements, the wide distribution of skin tones, and the objective measurement of skin tone using spectrophotometry. Sophisticated statistical modelling approaches were used to assess the performance of the pulse oximeters across several domains of measurement and diagnostic accuracy. We mitigated the impact of noise in skin tone measurement by deriving ITA from four L* and b* measurements for each patient, with little residual noise in the resulting average.

However, the study does have some limitations. To enable a gold standard measurement for comparison and to explore accuracy in those with low SaO_2_, the study enrolled patients who were critically ill. Other elements of critical illness that we were unable to account for in analyses may have affected the results. In particular, patients who are critically ill may be prone to poor perfusion of their digits owing to hypotension, hypovolaemia, or the use of vasopressor treatment. Poor perfusion has been shown to exacerbate the skin tone related bias of pulse oximetry.^21^ This effect could reduce the generalisability of the findings to a community based setting where these pulse oximeters are designed to be used. However, as the calculation of ITA does not include the a* values most directly affected by cutaneous blood flow, it is unlikely that skin tone values in this study were affected by perfusion. It is possible that differences between ITA measurements taken from the dorsal surface of the hand could differ slightly from those at the fingertip where pulse oximeters were placed. Finally, while the range of oxygen saturations observed covered the key decision thresholds in current treatment guidelines for home use, only 16% of SaO_2_ values were 92% or lower and only 3% were less than 88%.

### Implications for clinical practice

Healthcare providers need to remember that pulse oximetry is a way of estimating SaO_2_ that has several limitations and that performance can vary considerably between models of pulse oximeter; it is their responsibility to understand the performance characteristics of the device they are using. Manufacturers should ensure robust testing of devices before marketing them and continue to monitor performance after marketing. All performance data should be made available to end users.

The findings of our study and the work that preceded it need to be considered when determining the clinical relevance of SpO_2_ readings and steps taken to mitigate any potential harm that could occur by failing to appreciate the effect of skin tone on pulse oximetry accuracy. SpO_2_ readings should be interpreted in the context of other clinical information and trends in SpO_2_ values given greater importance than single readings, particularly in patients with darker skin tones. False negative readings translate to a failure to detect hypoxaemia, which could have important clinical ramifications, while false positive readings may result in the unnecessary administration of oxygen and other treatments. Healthcare systems should develop guidance to inform and help practitioners, patients, and the public, particularly in settings where additional clinical readings from other medical measurement devices would not be available. Because the accuracy of pulse oximetry varies between devices, across skin tones, and is dependent on SaO_2_, it is not possible to generate unified clinical guidance from our study findings.

### Implications for standards and regulation

The current International Organisation for Standardization (ISO) standard on basic safety and essential performance of pulse oximeters requires observations from only 10 people, with no requirements related to skin tone.^22^ This standard relies on a single performance metric, A_RMS_, which our study has shown is insensitive to potentially clinically important variations in performance by skin tone (and would be similarly insensitive to other biases of a comparable magnitude). This ISO standard is currently in the process of being reviewed and revised. In the UK, the Medicines and Healthcare products Regulatory Agency does not approve medical devices directly. Market approval is expected to include compliance with the ISO standard, but this is not mandatory.

The US Food and Drug Administration has recently published draft guidance aimed at improving the accuracy of pulse oximeters used in healthcare settings.^23^ The draft guidance recommends pulse oximeters are evaluated in 150 or more participants with a diverse range of skin tones, assessed by the Monk Skin Tone scale and ITA, and 20-24 paired measurements of SpO_2_ and SaO_2_ for each participant. In addition to A_RMS_, the draft guidance includes recommended success criteria in terms of variation in the bias across Monk Skin Tone categories and by ITA. Although the draft guidance goes some way to addressing the issues identified in our study, a 1.5 percentage point variation in bias across a 100° range of ITA would be considered non-disparate performance for SaO_2_ values in the range we studied. This is at the upper end of the variation in bias seen in our study, which translated into substantial variation in diagnostic accuracy. Because SpO_2_ thresholds may be used to guide clinical practice or healthcare seeking behaviour, more stringent criteria may need to be considered.

### Implications for further research

This study aimed to evaluate the accuracy of fingertip pulse oximeters designed to be used at home at oxygen saturations in the range relevant to clinical decision making in the community. Therefore, the findings are not directly generalisable to high fidelity pulse oximeters used in secondary care settings or to lower oxygen saturations relevant to clinical decision making in these settings. Future research should investigate whether the signals detected in hospital based retrospective studies are replicated using objective measures of skin tone. Our study has highlighted the complexity of the effect of skin tone on pulse oximeter accuracy and should serve as a framework to generate further research questions aiming to reduce bias in pulse oximetry. No gold standard method exists for measuring skin tone.^9^ We used spectrophotometry to generate ITA values to avoid the well described limitations of colour charts and the Fitzpatrick skin type,^24^ and took measurements from the dorsal surface of the hand because this is close to the fingertip. ITA values vary according to where on the body the measurements are taken,^8^ and further work needs to be undertaken to determine which site provides the most relevant values to optimise pulse oximetry. Additionally, in settings where spectrophotometry is not possible, reliable alternatives need to be evaluated.

### Conclusions

For any given SaO_2_ across the range studied, the five fingertip pulse oximeters evaluated gave SpO_2_ readings on average 0.6-1.5 percentage points higher for a patient with a median dark skin tone than for a patient with a median light skin tone. These small variations in bias translated into substantial differences in false positive and false negative rates for detecting hypoxaemia.

**What is already known on this topic**

Pulse oximeters measure peripheral haemoglobin oxygen saturation (SpO_2_) and were developed to identify hypoxaemia (low blood oxygen levels), but their accuracy is known to be affected by several factors
Combining evidence from previous studies indicates that darker skin tones, often assessed using ethnicity as a proxy for skin tone, are associated with overestimation of arterial haemoglobin oxygen saturation (SaO_2_)
Heterogeneity in study designs including a lack of objective skin tone measurement has prevented quantification of the impact of skin tone on the measurement and diagnostic accuracy of pulse oximeters

**What this study adds**

The five pulse oximeters assessed all gave higher SpO_2_ readings for patients with darker skin tones than for patients with lighter skin tones
Although absolute differences in readings were small, they can result in substantially higher rates of false negatives and lower rates of false positives in the diagnosis of hypoxaemia
Neither bias nor accuracy root mean square (the measure used to define manufacturing standards for pulse oximeter accuracy) adequately reflected differences in diagnostic accuracy across skin tones

## Data Availability

Deidentified participant data and associated documentation will be made available to researchers for any reasonable purpose, subject to a data use agreement, after completion and publication of all preplanned analyses. To request access to data, please visit: https://www.icnarc.org/data-services/access-our-data/. Full analysis code can be viewed at https://github.com/ICNARC/EXAKT

## Web extra

Extra material supplied by authors

Web appendix: Supplementary appendix

## Notes

EXAKT Study Investigators: Katie Sweet, Daniel Hill, Linda Pipira, Oluwatosin Komolafe, Marcela P Vizcaychipi, Sanjeet Chana, Laura Martins, Ilhan Wardhere, Dhinesh Sundaran, Laszlo Hollos, Anna Williams, David Golden, Rebecca Seaman, Miriam Davey, Laura Kent, Nazrudeen Ali, Ibrahim Nasr, Dee Durrant, Bincy Kariyadil, Brendan Sloan, Sarah Buckley, Amy Major, Mathew Smith, Dumindu Karangoda, Estefania Treus, Georgina Luck, Shilpa Gurung, Mandeep K Phull, Nauman Hussain, Tatiana Pogreban, Aparna George; Nicholas Truman, Qasim T Ahmed, Victoria Cunliffe, Aayesha Kazi, Mark De Neef, Poh Choo Teoh, Glykeria Pakou, Amitaa Maharajh, Sarah Penkett, Muthukumaran Gourishankar, Paul Gill, Roxanne Gray, Thomas Billyard, Carl Hawkins, Brendan B Spooner, Geraldine Ward, Monica Popescu, Theodora Christodoulopoulou, Amrinder Sayan, Jamie Gonzales, Redmond P Tully, Joy Dearden, Michelle Mulcahy, Carrole Blessen, Andrew Achilleos, Rosaleeta Reece-Anthony, Christine B Manalo, Kay Ellen Spikes, Ahilanandan Dushianthan, Michael Carter, Karen Salmon, Rachel Burnish, Christopher Hebbes, Joanna Shak, Megha Mathews, Navneet Ghuman, Alex Mattin, Geraldine Hambrook, Tom O Lawton, Omar Jundi, Sharwend Supermanian, Louise T Akeroyd, Thaventhran Prabhahar, Dhanalakshmi Bakthavatsalam, Suhieb Alhourani, Leena John, Mike Dean, Peter Mathews, Tabassum Khan; Lakshmi Aneesh, Dagan Lonsdale, Susannah Leaver, Sarah Farnell-Ward, Deborah Dawson, Nikki Yun, Maria Thanasi, Shreeja Dangol, Massimiliano Valcher, Angelo Rocha, Milafe Nimer, Frenita Dsouza, Rebecca Kanu, Maria Maiz Cordoba, Vincent Ventura, Edna Fernandes, Karen Lloyd, John Adam, Rohit Saha, Tom Williams, Kevin O'Reilly, Anna Broderick.

Contributors: DSM, DG, JCD, CM, OO, EH, KR, PM, and DAH conceptualised and designed the study; DSM, DG, CM, OO, EH, KR, PM, and DAH obtained funding for the study; DSM, JCD, DG, TS, AC, WNC, AFJ, RG, CM, OO, EH, KR, PM, and DAH conducted the study; TS and AFJ were responsible for project administration; JCD, AC, WNC, and DAH were responsible for data curation; JCD, AC, WNC, and DAH were responsible for study software; JCD, AC, WNC, and DAH undertook formal analysis of the data; DSM, JCD, DG, AC, WNC, EH, KR, PM, and DAH validated the findings; JCD, AC, WNC, and DAH created the visualisations; DSM, JCD, DG, KR, PM, and DAH wrote the original draft of the manuscript; DSM, JCD, DG, TS, AC, WNC, AFJ, RG, CM, OO, EH, KR, PM, and DAH reviewed and edited versions of the manuscript. JCD, AC, and DAH directly accessed and verified the underlying data reported in the manuscript. DSM is the guarantor. The corresponding author attests that all listed authors meet authorship criteria and that no others meeting the criteria have been omitted. All authors revised the manuscript and approved the final manuscript as submitted.

Transparency: The lead author (the manuscript’s guarantor) affirms that this manuscript is an honest, accurate, and transparent account of the study being reported; that no important aspects of the study have been omitted; and that any discrepancies from the study as planned (and, if relevant, registered) have been explained.

Dissemination to participants and related patient and public communities: We will initially disseminate the findings of our study to the public via our public website, using plain language. We are working with community groups who have assisted us throughout the study to produce infographics, animations, and plain language summaries specifically for members of the public who may be particularly affected by the findings. These will form the next phase of our public dissemination programme. We have already begun to engage with key healthcare and policy stakeholders in the UK to communicate our findings to them. We will continue to work with these stakeholders after the publication of this manuscript to facilitate future research and clinical guidance.

Provenance and peer review: Not commissioned; externally peer reviewed.
