# Supplementary material for: The impact of skin tone on performance of pulse oximeters used by NHS England COVID Oximetry @home scheme: measurement and diagnostic accuracy study
Source: BMJ. 2026 Jan 14;392:e085535. doi: 10.1136/bmj-2025-085535 (PMC12801414; doi:10.1136/bmj-2025-085535)
Supplement: Supplementary file 1 — Web appendix: Supplementary appendix [file mard085535.ww.pdf]

The impact of skin tone on the measurement and diagnostic accuracy of pulse oximeters used by the National Health Service COVID oximetry @home scheme

Supplementary appendix

Contents

Contents 1
List of tables 1
List of figures 1
EXAKT study investigators 2
Supplementary methods 2
  UK-ROX trial participant eligibility criteria 2
  Research without prior consent model 2
  Detailed methods for measurements using pulse oximeters 3
  List of blood gas co-oximeter machines in use at sites 3
  Detailed methods for measurement of skin tone 3
  Detailed methods for statistical analysis 4
References 6
Supplementary tables and figures 7

List of tables

Supplementary table 1 Patient characteristics and arterial blood gas observations, by skin tone group 7
Supplementary table 2 Bias in SpO_2 as a measure of SaO_2, by skin tone 8
Supplementary table 3 Precision of SpO_2 measurements, by skin tone 9
Supplementary table 4 Accuracy of SpO_2 as a measure of SaO_2 (bias + precision), by skin tone 10
Supplementary table 5 False negative rate of two SpO_2 thresholds for predicting $SaO_2 \leq 92\%$, by skin tone 11
Supplementary table 6 False positive rate of two SpO_2 thresholds for predicting $SaO_2 \leq 92\%$, by skin tone 12
Supplementary table 7 Area under the receiver operating characteristic curve of SpO_2 for predicting $SaO_2 \leq 92\%$, by skin tone 13
Supplementary table 8 Occult hypoxaemia by skin tone 14

List of figures

Supplementary figure 1 Recruitment flow diagram 15
Supplementary figure 2 Distribution of individual typology angle (ITA) in the EXAKT study cohort 16
Supplementary figure 3A Relationship between SpO_2 and SaO_2, by skin tone category (pulse oximeter A) 17
Supplementary figure 3B Relationship between SpO_2 and SaO_2, by skin tone category (pulse oximeter B) 18
Supplementary figure 3C Relationship between SpO_2 and SaO_2, by skin tone category (pulse oximeter C) 19
Supplementary figure 3D Relationship between SpO_2 and SaO_2, by skin tone category (pulse oximeter D) 20
Supplementary figure 3E Relationship between SpO_2 and SaO_2, by skin tone category (pulse oximeter E) 21
Supplementary figure 4 Relationship between bias and skin tone at different levels of SaO_2 22
Supplementary figure 5 Relationship between precision and skin tone at different levels of SaO_2 23
Supplementary figure 6 Relationship between accuracy and skin tone at different levels of SaO_2 24
Supplementary figure 7 Heat maps showing variation in bias, precision and accuracy across combinations of skin tone and SaO_2 25
Supplementary figure 8 False negative rate and false positive rate of $SpO_2 \leq 94\%$ for predicting $SaO_2 \leq 92\%$ 26

EXAKT study investigators

Bristol Royal Infirmary: Katie Sweet, Daniel Hill, Linda Pipira, Oluwatosin Komolafe; Chelsea and Westminster Hospital: Marcela P Vizcaychipi, Sanjeet Chana, Laura Martins, Ilhan Wardhere; Kettering General Hospital: Dhinesh Sundaran, Laszlo Hollos, Anna Williams; Maidstone Hospital: David Golden, Rebecca Seaman, Miriam Davey, Laura Kent; Northampton General Hospital: Nazrudeen Ali, Ibrahim Nasr, Dee Durrant, Bincy Kariyadil; Pinderfields Hospital: Brendan Sloan, Sarah Buckley, Amy Major, Mathew Smith; Queen Elizabeth Hospital, Woolwich: Dumindu Karangoda, Estefania Treus, Georgina Luck, Shilpa Gurung; Queen's Hospital, Romford: Mandeep K Phull, Nauman Hussain, Tatiana Pogreban, Aparna George; Royal Blackburn Hospital: Nicholas Truman, Qasim T Ahmed, Victoria Cunliffe, Aayesha Kazi; Royal Free Hospital: Mark De Neef, Poh Choo Teoh, Glykeria Pakou, Amitaa Maharajh; Tameside General Hospital: Sarah Penkett, Muthukumaran Gourishankar, Paul Gill, Roxanne Gray; University Hospital Coventry: Thomas Billyard, Carl Hawkins, Brendan B Spooner, Geraldine Ward; West Middlesex University Hospital: Monica Popescu, Theodora Christodoulopoulou, Amrinder Sayan, Jamie Gonzales; Royal Oldham Hospital: Redmond P Tully, Joy Dearden, Michelle Mulcahy, Carrole Blessen; University Hospital Lewisham: Andrew Achilleos, Rosaleeta Reece-Anthony, Christine B Manalo, Kay Ellen Spikes; University Hospital Southampton: Ahilanandan Dushianthan, Michael Carter, Karen Salmon, Rachel Burnish; Leicester Royal Infirmary: Christopher Hebbes, Joanna Shak, Megha Mathews, Navneet Ghuman; Stoke Mandeville Hospital: Alex Mattin, Geraldine Hambrook, Tom O Lawton; Bradford Royal Infirmary: Omar Jundi, Sharwend Supermanian, Louise T Akeroyd; North Middlesex University Hospital: Thaventhran Prabhahar, Dhanalakshmi Bakthavatsalam, Suhieb Alhourani, Leena John; Northwick Park Hospital: Mike Dean, Peter Mathews, Tabassum Khan; Lakshmi Aneesh; St George's Hospital: Dagan Lonsdale, Susannah Leaver, Sarah Farnell-Ward, Deborah Dawson, Nikki Yun, Maria Thanasi, Shreeja Dangol, Massimiliano Valcher, Angelo Rocha, Milafe Nimer, Frenita Dsouza, Rebecca Kanu, Maria Maiz Cordoba, Vincent Ventura, Edna Fernandes, Karen Lloyd, John Adam; Kings College Hospital and Princess Royal University Hospital: Rohit Saha, Tom Williams, Kevin O'Reilly, Anna Broderick.

Supplementary methods

UK-ROX trial participant eligibility criteria

Inclusion criteria

1. Aged ≥ 18 years
2. Receiving invasive mechanical ventilation in the ICU following an unplanned ICU admission OR invasive mechanical ventilation started in the ICU
3. Receiving supplemental oxygen (fractional inspired concentration oxygen (F_IO_2) > 0.21) at the time of enrolment

Exclusion criteria

1. Previously randomised into UK-ROX in the last 90 days
2. Currently receiving extracorporeal membrane oxygenation (ECMO)
3. The treating clinician considers that one trial intervention arm is either indicated or contraindicated

Research without prior consent model

Two routes of entry into the EXAKT study were possible:

1. Patients already enrolled into the UK-ROX trial

At the point where a patient was randomised into the UK-ROX trial, the site was notified whether the patient could also be enrolled into the EXAKT study following the research without prior consent (RWPC) model already employed in the UK-ROX trial.[1] The consent process was incorporated into the existing consent model for the UK-ROX trial. Following randomisation, permission was sought to continue collecting data for the EXAKT study from a Personal Consultee (i.e., relative or close friend), Nominated Consultee (e.g., Independent Mental Capacity Advocate appointed by the NHS Hospital Trust or an independent doctor, i.e., not involved in the trial), and/or the patient themselves. As part of the RWPC conversation for the main UK-ROX trial, consultees and/or patients were asked for consent for the data collected as part of the EXAKT study. In the rare situation where a patient was deemed by the treating clinical team to

have full capacity and was able to give informed consent at the point of meeting the eligibility criteria, they were approached directly prior to randomisation into the main UK-ROX trial and for inclusion into the study. No data for the EXAKT study was entered into the study database prior to agreement from the Consultee or patient.

2. Patients screened and not randomised into the UK-ROX trial

For patients screened and not randomised into the UK-ROX trial but deemed eligible for the EXAKT study, agreement was obtained from the patient if they have full mental capacity or from a Personal or Nominated Consultee prior to the patient being enrolled into the EXAKT study.

Detailed methods for measurements using pulse oximeters

The finger-tip pulse oximeters under evaluation were placed onto the patient's fingers on the same hand (ensuring nail varnish has been removed from the patient's finger prior to this), with the screen displaying the SpO_2 on the dorsal surface of the finger, according to NHS England advice to patients using pulse oximeters at home (https://www.england.nhs.uk/nhs-at-home/covid-oximetry-at-home/). Once both SpO_2 values had stabilised they were recorded.

List of blood gas co-oximeter machines in use at sites

- GEM Premier 4000 (Instrumentation Laboratory, Bedford, MA, United States)
- GEM Premier 5000 (Instrumentation Laboratory, Bedford, MA, United States)
- ABL90 Flex Plus (Radiometer, Copenhagen, Denmark)
- ABL800 Flex (Radiometer, Copenhagen, Denmark)
- Cobas b 221 (Roche Diagnostics, Mannheim, Germany)
- Rapidpoint 500/500e (Siemens Healthcare, Forcheim, Germany)

Detailed methods for measurement of skin tone

Skin tone of each participant was measured using a Konica Minolta CM-600d spectrophotometer with the target mask and stabilising plate attached. The circular measuring aperture of the CM-600d is 8 mm diameter, thus each single skin tone measurement area was $50.24mm^2$ (i.e. $\pi \times 4^2$ mm^2), and as four separate areas of skin were assessed for each participant, this equated to a total skin tone measurement area of 2 cm^2 per participant.

Prior to commencing the study, operators at all study sites were trained by a member of the research team on how to measure skin tone, accompanied by information on skin pigmentation (including cutaneous melanin, CIELAB (L*, a*, b*), how the spectrophotometer works, individual typology angle (ITA), and ITA values for different skin tone groups). In addition, all operators were provided with a standard operating procedure in booklet format and online PDF, and an EXAKT study video on measuring skin tone. The standard operating procedure and video were generated by Konica Minolta, Warrington, UK in collaboration with the project skin tone lead. Operators were also instructed to contact the skin tone lead investigator if they experienced any problems with the spectrophotometer and/or if any of the measured L*, a* and b* values seemed to differ from that which would be expected according to the visible colour of the participant's skin.

The spectrophotometer was calibrated before each use (each time it was switched on), using the zero-calibration tube and white calibration tile. A 70% alcohol sterile wipe was employed to clean the device and aperture stabilising plate between each patient. The operator ensured that the configuration line of the display screen exhibited SCE 10° / D65 and used sample mode to record skin tone measurements, thus ensuring that all measurements of skin tone throughout the study were recorded in the memory chip of the spectrophotometer. Prior to each new participant, a 'base zero' was recorded by pointing the measurement aperture of the spectrophotometer into 1m of space towards the floor. This 'base zero' value simplified finding the first measurement of a dataset for the relevant participant because it was easily distinguishable from the skin tone measurement data, thus ensuring that the correct values for each participant were recorded in the case report form.

Skin tone was quantified on four separate sites on the dorsum of the participant's non-dominant (left if unknown) hand, while the hand and forearm were in a horizontal position which was supported (e.g. on the bed) to avoid any movement

during the procedure. The operator also made sure to avoid any pigmented lesions (moles, lentigos, etc.), tattoos, skin disease, visible veins, bruises, or other skin problems so that skin tone measurements were taken only on normal skin. In (occasional) cases where the operator considered it was not possible to take accurate skin tone measurements on the non-dominant hand; the measurements were taken of normal skin on the dominant hand. Following skin tone measurement, the participant's L*, a* and b* values were recorded on the case report form for that patient. The L* and b* values were used to calculate the ITA [arctan ((L* − 50)/b*) × (180/π)], used as an objective continuous measure of skin tone within the statistical models. Previously defined categories of skin tone based on ITA were used in descriptive results (very light, ITA ≥ 55°; light, 41° ≤ ITA < 55°; intermediate, 28° ≤ ITA < 41°; tan, 10° ≤ IT < 28°; brown, −30° ≤ ITA < 10°; dark, ITA < −30°).[2]

Detailed methods for statistical analysis

Formula for bias

Mean bias was calculated in accordance with BS EN ISO 80607261:2019, Annex CC[3]:

$$B = \frac{\sum_{i=1}^{n} (SpO_{2i} - S_{Ri})}{n} = \frac{\sum_{i=1}^{n} \left[(SpO_{2i} - SpO_{2fit,i}) + (SpO_{2fit,i} - S_{Ri}) \right]}{n} = 0 + \frac{\sum_{i=1}^{n} b_i}{n} \qquad \text{(CC.3)}$$

where S_R represents the reference value of SaO_2 and SpO_{2fit} represents the fitted value predicted by the multivariable fractional probit regression model of SpO_2 on SaO_2 plus covariates (as described below). The first form of equation CC.3 represents the formula for unadjusted estimates and the second form represents the formula used for adjusted estimates.

Formula for precision

BS EN ISO 80607261:2019, Annex CC recommends the following formula for calculation of precision:

$$s_{res} = \sqrt{\frac{\sum_{i=1}^{n} \left(SpO_{2i} - SpO_{2fit,i} \right)^2}{(n-2)}} \qquad \text{(CC.4)}$$

where SpO_{2fit} represents a fitted value. For unadjusted estimates of precision, $SpO_{2fit,i}$ represents the simple local mean of SpO_2 conditional on SaO_2 only. For adjusted estimates, $SpO_{2fit,i}$ represents the fitted values obtained from the SpO_2 regression model in described for bias, above. To support regression modelling of the relationship between precision, saturation and skin tone, the squared residual from the SpO_2 model, $\left(SpO_{2i} - SpO_{2fit,i}\right)^2$, was used as the dependent variable in a second regression model with identical covariate structure. Adjusted estimates of precision represent the mean fitted values predicted by this two-stage model, an approximation of the definition recommended by BS EN ISO 80607-2-61:2019 which ignores the "minus 2" degrees of freedom in the denominator:

$$\widehat{s_{res}} = \sqrt{\frac{\sum_{i=1}^{n} \left(SpO_{2i} - SpO_{2fit,i} \right)^2}{n}}$$

The difference between s_{res} and $\widehat{s_{res}}$ can be expected to be negligible except in small samples (e.g. $n < 20$). To ensure comparability of raw and adjusted results, $\widehat{s_{res}}$ was used for both.

Formula for accuracy root mean square

Accuracy root mean square, A_{rms}, was calculated in accordance with BS EN ISO 80607-2-61:2019, Annex CC:

$$A_{\mathrm{rms}} = \sqrt{\frac{\sum_{i=1}^{n}\left(SpO_{2i} - S_{\mathrm{R}i}\right)^2}{n}} \qquad \text{(CC.5)}$$

To support regression modelling of the relationship between accuracy, saturation and skin tone, the squared measurement error, $(SpO_{2i} - S_{\mathrm{R}i})^2$, was used as the dependent variable in a regression model with identical covariate structure as the models described above, and results presented as the square root of the mean fitted value.

Statistical modelling

For each accuracy measure, a single fractional probit regression model was fitted to the set of complete observations, adjusted for fixed effects of pulse oximeter model, ITA, SaO_2 and haemoglobin concentration, and interactions between pulse oximeter model and ITA, pulse oximeter model and SaO_2, and ITA and SaO_2. ITA, SaO_2 and haemoglobin concentration were modelled continuously using restricted cubic splines with four knots placed at the 5th, 35th, 65th and 95th percentiles of the distributions.[4] The influence of knot positions for SaO_2 on the shape of the resulting curves was explored in sensitivity analyses. Interactions between ITA and SaO_2 were modelled by including interaction terms between the linear component of each covariate and all restricted cubic spline base variables of the other covariate.[4] To analyse bias and the impact of skin tone on bias, a model was fitted with SpO_2 as the dependent variable. Bias was then derived as the difference between the marginal predicted SpO_2 and the observed SaO_2. To analyse precision and the impact of skin tone on precision, a model was fitted with the squared deviation between the SpO_2 and the marginal predicted SpO_2 from the bias model as the dependent variable. To analyse accuracy and the impact of skin tone on accuracy, a model was fitted with the squared difference between the SpO_2 and paired SaO_2 as the dependent variable. Because methods have not yet been identified for including random effects in fractional regression models, fixed effects of site were included to account for clustering of patients within sites, an approach that yields consistent estimation with conservative standard errors. To account for repeated measures within patients, the variance-covariance matrices were clustered at the patient level.

To estimate false negative and false positive rates, multilevel logistic regression models were fitted with binary dependent variables of $SpO_2 \leq 92\%$ and $SpO_2 \leq 94\%$. The models included the same fixed effects as the regression models for accuracy, and random effects for patients nested within sites. The false negative rate (one minus sensitivity) of $SpO_2 \leq 92\%$ or 94% to predict $SaO_2 \leq 92\%$ was estimated as the marginal predicted probability of $SpO_2 > 92\%$ or 94% from the respective model averaged over the distribution of SaO_2 among patients with $SaO_2 \leq 92\%$. Similarly, the false positive rate (one minus specificity) of $SpO_2 \leq 92\%$ or 94% to predict $SaO_2 \leq 92\%$ was estimated as the marginal predicted probability of $SpO_2 \leq 92\%$ or 94% from the respective model averaged over the distribution of SaO_2 among patients with $SaO_2 > 92\%$. The area under the ROC curve was estimated using a probit ROC regression model with a dependent variable of $SaO_2 \leq 92\%$, independent variable of SpO_2 and adjusted for fixed effects of pulse oximeter model and ITA, and interactions between pulse oximeter model and ITA, and with control covariates of haemoglobin concentration and the variance-covariance matrix clustered at the patient level. The rate of occult hypoxaemia was estimated as the marginal predicted probability of $SpO_2 > 92\%$ from the multilevel logistic regression model for $SpO_2 \leq 92\%$, averaged over the distribution of SaO_2 among patients with $SaO_2 < 88\%$.

Of note, the restricted range of SaO_2 (0–100%) imposes substantial challenges to the presentation and interpretation of all measures of pulse oximeter accuracy. As SaO_2 approaches 100%, any error in the prediction of SaO_2 using SpO_2 becomes increasingly restricted to negative values (e.g. SpO_2 cannot overestimate an SaO_2 of 100%). Both bias and precision are therefore expected to vary across the range of SaO_2, and any summary measures of error or bias must be conditioned on a given distribution of SaO_2 such as the distribution observed in the EXAKT study cohort, which may be quite different from the distribution for any given external population. Similarly, sensitivity and specificity are conditional on the observed distribution of SaO_2 among patients with and without hypoxaemia, respectively, regardless of the threshold used to define hypoxaemia. Results are therefore presented both graphically, over the full spectrum of SaO_2 that was available for analysis, and as tabulated summary statistics that are conditional on the distribution of SaO_2 observed in the EXAKT study cohort. Attention should be focused on graphical results and strong caution exercised when generalising summary statistics beyond the EXAKT cohort; differences in the distribution of SaO_2 in any external population should be expected to imply differences in accuracy measures. However, patterns of variation in accuracy across skin tones, the focus of this analysis, are likely to be generalisable.

References

1. Martin DS, Shahid T, Gould DW, et al. Evaluating the clinical and cost-effectiveness of a conservative approach to oxygen therapy for invasively ventilated adults in intensive care: Protocol for the UK-ROX trial. *J Intensive Care Soc* 2024; **25**(2): 223-30.
2. Chardon A, Cretois I, Hourseau C. Skin colour typology and suntanning pathways. *Int J Cosmet Sci* 1991; **13**(4): 191-208.
3. The British Standards Institution. BS EN ISO 80607-2-61:2019: Medical electrical equipment Part 2-61: Particular requirements for basic safety and essential performance of pulse oximeter equipment (ISO 80601-2-61:2017, Corrected version 2018-02). London: British Standards Institution; 2019.
4. Harrell FE, Jr. Regression modeling strategies: with applications to linear models, logistic regression, and survival analysis. New York: Springer; 2001.

Supplementary tables and figures

Supplementary table 1 Patient characteristics and arterial blood gas observations, by skin tone group

	Skin tone group				
	Very light or light [1]	Intermediate	Tan	Brown	Dark
**Patient characteristics**					
N	111	139	198	305	150
Age [2], mean (SD) [N]	56.4 (17.8) [108]	58.1 (16.4) [131]	55.4 (17.1) [184]	55.1 (15.2) [284]	53.6 (14.2) [141]
Male [2], n (%) [N]	51 (47.2%) [108]	88 (67.2%) [131]	132 (71.7%) [184]	189 (66.5%) [284]	106 (75.2%) [141]
Ethnic group [3], n (%)	[N = 91 (82.0%)]	[N = 113 (81.3%)]	[N = 162 (81.8%)]	[N = 251 (82.3%)]	[N = 120 (80.0%)]
White	76 (83.5%)	72 (63.7%)	49 (30.2%)	28 (11.2%)	7 (5.8%)
Asian	6 (6.6%)	24 (21.2%)	74 (45.7%)	104 (41.4%)	19 (15.8%)
Black	1 (1.1%)	0 (0.0%)	13 (8.0%)	85 (33.9%)	78 (65.0%)
Mixed or Other	8 (8.8%)	17 (15.0%)	26 (16.0%)	34 (13.5%)	16 (13.3%)
Chronic respiratory disease [4], n (%) [N]	11 (11.0%) [100]	8 (6.6%) [121]	11 (6.8%) [161]	14 (5.9%) [239]	5 (4.1%) [123]
Reason for admission to critical care (body system) [1], n (%)	[N = 108 (97.3%)]	[N = 131 (94.2%)]	[N = 184 (92.9%)]	[N = 284 (93.1%)]	[N = 141 (94.0%)]
Respiratory	33 (30.6%)	42 (32.1%)	47 (25.5%)	82 (28.9%)	46 (32.6%)
Cardiovascular	22 (20.4%)	29 (22.1%)	37 (20.1%)	58 (20.4%)	23 (16.3%)
**Arterial blood gas observations**					
N	674	868	1213	1987	914
SaO_2, %, median (IQR)	95 (93, 97)	96 (94, 97)	96 (94, 97)	96 (94, 98)	96 (94, 98)
SaO_2 range [5], n (%)					
< 88%	9 (1.3%)	13 (1.5%)	19 (1.6%)	39 (2.0%)	15 (1.6%)
88% – 92%	115 (17.1%)	129 (14.9%)	161 (13.3%)	265 (13.3%)	126 (13.8%)
93% – 94%	151 (22.4%)	151 (17.4%)	204 (16.8%)	289 (14.5%)	145 (15.9%)
> 94%	399 (59.2%)	575 (66.2%)	829 (68.3%)	1394 (70.2%)	628 (68.7%)
Haemoglobin, g/L, median (IQR)	104 (89, 119)	104 (89, 120)	99 (85, 115)	99 (85, 117)	98 (87, 114)
Carboxyhaemoglobin [6], %, median (IQR)	1.1 (0.8, 1.4)	1.1 (0.8, 1.5)	1.2 (0.8, 1.5)	1.2 (0.9, 1.5)	1.2 (0.8, 1.5)
Methaemoglobin [7], %, median (IQR)	0.7 (0.4, 1.0)	0.7 (0.3, 1.1)	0.7 (0.4, 1.1)	0.7 (0.5, 1.0)	0.7 (0.5, 1.1)

[1] Due to small numbers, “very light” (N=13) is combined with “light” in tables and figures.

[2] Based on linked routine data. N=55 (6.1%) not linked.

[3] Based on linked routine data. N=55 (6.1%) not linked, N=111 (12.3%) recorded as “Not stated”.

[4] Shortness of breath with light activity due to pulmonary disease and evident within the six months prior to admission. Based on linked routine data. N=55 (6.1%) not linked, N=104 (11.5%) missing.

[5] Rounded to nearest percentage point, for consistency with SpO_2.

[6] N=36 (0.6%) missing.

[7] N=71 (1.3%) missing.

Supplementary table 2 Bias in SpO_2 as a measure of SaO_2, by skin tone

	Pulse oximeter					Overall
	A	B	C	D	E	
Observed mean error (mean SpO_2 – mean SaO_2) [1]						
Very light or light	−1.9 (93.5 – 95.3)	−3.4 (91.1 – 94.5)	−1.4 (94.2 – 95.5)	0.2 (95.0 – 94.7)	−2.6 (92.9 – 95.5)	−1.8 (93.2 – 95.0)
Intermediate	−1.4 (93.6 – 95.1)	−3.0 (91.6 – 94.7)	−1.1 (94.3 – 95.4)	0.7 (95.9 – 95.2)	−1.9 (93.7 – 95.6)	−1.4 (93.8 – 95.2)
Tan	−1.7 (93.6 – 95.3)	−2.8 (92.3 – 95.0)	−0.8 (94.8 – 95.6)	0.9 (96.3 – 95.4)	−2.0 (93.2 – 95.2)	−1.3 (94.0 – 95.3)
Brown	−1.5 (94.0 – 95.5)	−2.0 (92.9 – 94.9)	−0.4 (95.1 – 95.5)	0.7 (96.3 – 95.6)	−1.5 (94.0 – 95.5)	−0.9 (94.5 – 95.4)
Dark	−1.1 (94.5 – 95.6)	−1.9 (93.1 – 95.1)	−0.2 (95.7 – 95.9)	1.1 (96.4 – 95.2)	−1.3 (94.2 – 95.5)	−0.6 (94.8 – 95.5)
Overall	−1.5 (93.9 – 95.4)	−2.5 (92.3 – 94.9)	−0.7 (94.9 – 95.6)	0.7 (96.1 – 95.3)	−1.8 (93.7 – 95.4)	−1.1 (94.2 – 95.3)
Adjusted mean error, cohort average (95% CI) [2]						
Very light or light	−1.7 (−2.0, −1.4)	−3.9 (−4.2, −3.6)	−1.1 (−1.4, −0.8)	0.1 (−0.2, 0.5)	−2.4 (−2.7, −2.1)	−1.8 (−1.9, −1.6)
Intermediate	−1.8 (−2.0, −1.5)	−3.5 (−3.7, −3.2)	−0.8 (−1.0, −0.6)	0.5 (0.3, 0.8)	−1.9 (−2.1, −1.7)	−1.5 (−1.6, −1.4)
Tan	−1.7 (−1.9, −1.4)	−3.0 (−3.3, −2.8)	−0.5 (−0.7, −0.3)	0.8 (0.6, 1.1)	−1.6 (−1.8, −1.3)	−1.2 (−1.3, −1.1)
Brown	−1.2 (−1.5, −1.0)	−2.4 (−2.8, −2.1)	−0.2 (−0.4, 0.0)	0.9 (0.6, 1.1)	−1.5 (−1.8, −1.2)	−0.9 (−1.0, −0.7)
Dark	−1.1 (−1.3, −0.8)	−2.4 (−2.9, −1.9)	0.1 (−0.1, 0.3)	1.1 (0.7, 1.5)	−1.2 (−1.6, −0.7)	−0.7 (−0.9, −0.5)
Overall	−1.4 (−1.6, −1.3)	−3.0 (−3.2, −2.8)	−0.4 (−0.5, −0.2)	0.7 (0.6, 0.9)	−1.6 (−1.8, −1.4)	−1.1 (−1.2, −1.0)
Difference between dark and light skin tones [3]	0.6 (0.2, 1.0)	1.5 (0.9, 2.1)	1.2 (0.8, 1.5)	1.0 (0.5, 1.5)	1.3 (0.8, 1.7)	1.1 (0.9, 1.3)
Observed mean error over $SaO_2 \leq 92\%$ (mean SpO_2 – mean SaO_2) [1]						
Very light or light	−1.4 (89.5 − 90.8)	−2.7 (87.7 − 90.4)	0.8 (90.9 − 90.1)	2.0 (92.4 − 90.4)	−2.0 (88.5 − 90.5)	−0.8 (89.7 − 90.5)
Intermediate	−0.1 (90.3 − 90.3)	−2.4 (88.1 − 90.5)	0.0 (90.4 − 90.4)	3.3 (93.3 − 89.9)	−0.6 (89.7 − 90.3)	−0.2 (90.1 − 90.3)
Tan	0.3 (90.5 − 90.2)	−2.2 (88.0 − 90.2)	0.4 (91.2 − 90.8)	3.4 (93.5 − 90.1)	−0.6 (89.5 − 90.1)	0.2 (90.5 − 90.3)
Brown	1.0 (91.2 − 90.2)	−0.8 (89.1 − 89.9)	0.4 (90.3 − 89.8)	3.6 (93.7 − 90.1)	−0.0 (90.0 − 90.0)	0.8 (90.8 − 90.0)
Dark	1.2 (91.1 − 89.9)	−0.5 (89.7 − 90.2)	1.4 (91.4 − 90.0)	3.3 (93.0 − 89.7)	1.6 (90.5 − 88.9)	1.3 (91.1 − 89.8)
Overall	0.4 (90.7 − 90.2)	−1.6 (88.6 − 90.2)	0.5 (90.7 − 90.2)	3.2 (93.2 − 90.1)	−0.2 (89.8 − 90.0)	0.4 (90.5 − 90.1)
Adjusted mean error over $SaO_2 \leq 92\%$ (95% CI) [2]						
Very light or light	0.2 (−0.5, 0.9)	−2.5 (−3.2, −1.9)	−0.4 (−1.2, 0.3)	2.1 (1.4, 2.8)	−1.4 (−2.1, −0.7)	−0.4 (−0.8, 0.0)
Intermediate	−0.0 (−0.7, 0.6)	−2.2 (−2.7, −1.6)	−0.1 (−0.7, 0.4)	2.6 (2.0, 3.2)	−0.9 (−1.5, −0.3)	−0.1 (−0.4, 0.2)
Tan	0.1 (−0.6, 0.7)	−1.5 (−2.1, −1.0)	0.4 (−0.2, 0.9)	3.1 (2.5, 3.6)	−0.3 (−0.9, 0.2)	0.4 (0.0, 0.7)
Brown	1.2 (0.7, 1.7)	−0.2 (−0.9, 0.5)	1.4 (0.9, 1.9)	3.5 (2.9, 4.1)	0.3 (−0.4, 0.9)	1.2 (0.9, 1.6)
Dark	0.9 (0.2, 1.5)	−0.8 (−1.5, 0.0)	1.2 (0.6, 1.9)	3.5 (2.6, 4.3)	0.2 (−0.6, 1.0)	1.0 (0.6, 1.5)
Overall	0.6 (0.1, 1.0)	−1.2 (−1.7, −0.8)	0.7 (0.2, 1.1)	3.1 (2.6, 3.5)	−0.3 (−0.8, 0.2)	0.6 (0.4, 0.8)
Difference between dark and light skin tones [3]	0.7 (−0.1, 1.5)	1.8 (0.8, 2.7)	1.7 (0.8, 2.5)	1.4 (0.5, 2.3)	1.6 (0.7, 2.5)	1.4 (0.8, 2.1)

Pulse oximeters: A, ChoiceMMed Oxywatch MD300C19; B, ChoiceMMed Oxywatch MD300C13-CC12; C, Creative Medical PC-60B1-BL; D, Biolight Meditech M70C; E, Medlinket AM802 NHE.

[1] Mean difference between SpO_2 and SaO_2 (bias).

[2] Mean difference between expected SpO_2 and SaO_2, with expected values predicted using a fractional probit regression of SpO_2 on SaO_2 and skin tone (individual typology angle, ITA), adjusted for pulse oximeter, haemoglobin and repeated observations within patients.

[3] Expected bias for a patient with median dark skin tone (−44°) minus the expected bias for a patient with median very light or light skin tone (46°), estimated using the model described above.

Supplementary table 3 Precision of SpO_2 measurements, by skin tone

	Pulse oximeter					Overall
	A	B	C	D	E	
Observed root mean squared error (95% CI) [1]						
Very light or light	2.6 (2.3, 2.9)	4.1 (3.3, 4.8)	2.2 (1.6, 2.9)	4.1 (2.7, 5.6)	2.7 (2.3, 3.2)	3.4 (2.9, 4.0)
Intermediate	3.6 (2.4, 4.7)	3.5 (2.9, 4.1)	2.5 (2.0, 3.1)	3.3 (2.1, 4.4)	2.7 (2.3, 3.0)	3.1 (2.7, 3.6)
Tan	3.0 (2.6, 3.3)	3.3 (2.9, 3.6)	2.4 (2.1, 2.8)	2.4 (2.1, 2.6)	3.4 (2.6, 4.1)	2.9 (2.7, 3.2)
Brown	3.6 (2.8, 4.5)	4.1 (3.4, 4.8)	2.4 (2.1, 2.7)	3.8 (2.8, 4.7)	3.5 (2.8, 4.2)	3.6 (3.1, 4.0)
Dark	2.9 (2.4, 3.3)	4.1 (2.4, 5.9)	2.0 (1.7, 2.4)	4.6 (2.3, 6.8)	5.1 (1.9, 8.4)	3.8 (2.8, 4.9)
Overall	3.3 (2.8, 3.7)	3.9 (3.5, 4.3)	2.3 (2.2, 2.5)	3.6 (3.0, 4.3)	3.6 (2.9, 4.3)	3.4 (3.1, 3.7)
Adjusted root mean squared error, cohort average (95% CI) [2]						
Very light or light	2.9 (2.4, 3.3)	3.8 (3.4, 4.3)	2.5 (2.1, 2.8)	3.8 (2.9, 4.7)	2.9 (2.5, 3.2)	3.2 (3.0, 3.5)
Intermediate	3.3 (2.8, 3.7)	3.5 (3.2, 3.9)	2.4 (2.1, 2.7)	3.1 (2.5, 3.7)	2.6 (2.2, 3.1)	3.0 (2.8, 3.2)
Tan	3.6 (3.0, 4.1)	3.4 (3.1, 3.7)	2.4 (2.1, 2.7)	2.8 (2.4, 3.3)	2.8 (2.2, 3.4)	3.0 (2.8, 3.2)
Brown	3.4 (2.9, 3.8)	3.9 (3.3, 4.5)	2.4 (2.1, 2.6)	3.7 (3.0, 4.4)	4.3 (3.2, 5.3)	3.6 (3.3, 4.0)
Dark	2.9 (2.5, 3.3)	4.7 (3.3, 6.1)	2.2 (2.0, 2.4)	4.3 (2.7, 5.8)	4.2 (2.0, 6.5)	3.8 (3.1, 4.5)
Overall	3.3 (3.0, 3.5)	3.9 (3.6, 4.2)	2.4 (2.2, 2.5)	3.6 (3.1, 4.2)	3.6 (2.9, 4.2)	3.4 (3.2, 3.6)
Difference between dark and light skin tones [3]	0.1 (−0.3, 0.4)	0.8 (−0.4, 2.0)	−0.3 (−0.6, 0.1)	0.4 (−0.7, 1.6)	1.3 (−0.6, 3.3)	0.6 (−0.1, 1.3)
Observed root mean squared error over $SaO_2 \leq 92\%$ (95% CI) [1]						
Very light or light	3.3 (2.2, 4.3)	4.5 (3.1, 5.8)	3.3 (1.4, 5.2)	4.1 (2.7, 5.6)	3.1 (2.3, 4.0)	3.9 (3.1, 4.7)
Intermediate	5.0 (1.6, 8.5)	3.2 (2.6, 3.9)	3.7 (1.9, 5.5)	3.0 (2.1, 3.8)	3.6 (2.3, 4.9)	3.8 (2.6, 4.9)
Tan	3.7 (3.0, 4.4)	3.2 (2.5, 3.8)	2.9 (2.1, 3.7)	3.6 (2.9, 4.3)	3.3 (2.2, 4.4)	3.4 (2.9, 3.8)
Brown	3.4 (2.7, 4.0)	5.5 (4.3, 6.8)	3.7 (2.9, 4.5)	3.7 (2.9, 4.4)	3.9 (3.0, 4.7)	4.2 (3.6, 4.8)
Dark	3.1 (2.2, 4.0)	3.5 (2.7, 4.2)	3.1 (2.3, 3.9)	6.2 (2.6, 9.9)	3.6 (1.6, 5.6)	4.1 (2.8, 5.3)
Overall	3.7 (2.9, 4.5)	4.4 (3.8, 5.0)	3.4 (2.9, 3.9)	4.2 (3.2, 5.1)	3.6 (3.0, 4.1)	3.9 (3.5, 4.3)
Adjusted root mean squared error over $SaO_2 \leq 92\%$ (95% CI) [2]						
Very light or light	3.4 (2.1, 4.7)	4.6 (3.8, 5.5)	3.6 (2.7, 4.5)	5.0 (3.5, 6.4)	3.8 (3.0, 4.6)	4.1 (3.5, 4.8)
Intermediate	4.1 (2.6, 5.5)	4.4 (3.7, 5.1)	3.7 (2.8, 4.6)	4.2 (3.1, 5.4)	3.6 (2.9, 4.4)	4.0 (3.4, 4.7)
Tan	4.4 (3.0, 5.8)	4.2 (3.5, 4.9)	3.6 (2.8, 4.5)	3.9 (2.9, 4.8)	3.8 (2.9, 4.8)	4.0 (3.4, 4.6)
Brown	3.7 (2.9, 4.4)	4.3 (3.3, 5.4)	3.2 (2.5, 3.8)	4.4 (3.6, 5.3)	5.2 (3.4, 7.1)	4.2 (3.6, 4.9)
Dark	3.6 (2.6, 4.6)	5.6 (4.2, 7.1)	3.3 (2.3, 4.2)	5.5 (3.8, 7.2)	5.6 (1.8, 9.3)	4.9 (3.6, 6.2)
Overall	3.9 (2.9, 4.8)	4.7 (4.1, 5.3)	3.4 (2.8, 4.0)	4.6 (3.9, 5.4)	4.6 (3.1, 6.1)	4.3 (3.8, 4.7)
Difference between dark and light skin tones [3]	0.2 (−1.2, 1.5)	1.0 (−0.7, 2.7)	−0.3 (−1.5, 0.9)	0.6 (−1.6, 2.7)	1.8 (−1.6, 5.1)	0.7 (−0.7, 2.2)

Pulse oximeters: A, ChoiceMMed Oxywatch MD300C19; B, ChoiceMMed Oxywatch MD300C13-CC12; C, Creative Medical PC-60B1-BL; D, Biolight Meditech M70C; E, Medlinket AM802 NHE.

[1] Root mean squared difference between observed SpO_2 and fitted SpO_2 using a fractional probit regression of SpO_2 on SaO_2 and skin tone (individual typology angle, ITA), adjusted for pulse oximeter, haemoglobin and repeated observations within patients (the same model used to estimate adjusted mean error in Table S1).

[2] Square root of the expected squared difference between observed SpO_2 and fitted SpO_2, where expected values were predicted using a second fractional probit regression of the squared difference between observed SpO_2 and fitted SpO_2 on the same variables as the first model, with identical model structure.

[3] Expected precision for a patient with median dark skin tone (−44°) minus the expected precision for a patient with median very light or light skin tone (46°), estimated using the two-stage model described above.

Supplementary table 4 Accuracy of SpO_2 as a measure of SaO_2 (bias + precision), by skin tone

	Pulse oximeter					Overall
	A	B	C	D	E	
Observed accuracy root mean square (95% CI) [1]						
Very light or light	3.2 (2.8, 3.6)	5.4 (4.5, 6.3)	2.8 (2.0, 3.5)	4.3 (2.9, 5.7)	3.8 (3.2, 4.3)	4.2 (3.6, 4.8)
Intermediate	4.0 (2.8, 5.1)	4.6 (3.9, 5.4)	2.8 (2.3, 3.3)	3.5 (2.5, 4.6)	3.3 (2.8, 3.7)	3.7 (3.3, 4.1)
Tan	3.6 (3.1, 4.0)	4.3 (3.8, 4.9)	2.7 (2.3, 3.1)	2.9 (2.5, 3.3)	4.0 (3.2, 4.7)	3.6 (3.3, 3.8)
Brown	4.2 (3.2, 5.1)	4.6 (3.9, 5.4)	2.5 (2.2, 2.8)	4.1 (3.3, 5.0)	3.9 (3.1, 4.6)	4.0 (3.5, 4.4)
Dark	3.3 (2.8, 3.8)	4.7 (2.9, 6.4)	2.2 (1.9, 2.6)	4.8 (2.9, 6.7)	5.5 (2.3, 8.7)	4.2 (3.2, 5.1)
Overall	3.8 (3.4, 4.2)	4.7 (4.3, 5.1)	2.6 (2.4, 2.8)	4.0 (3.4, 4.5)	4.1 (3.4, 4.7)	3.9 (3.7, 4.2)
Adjusted accuracy root mean square, cohort average (95% CI) [2]						
Very light or light	3.4 (3.0, 3.9)	5.2 (4.7, 5.7)	2.9 (2.5, 3.3)	4.0 (3.2, 4.8)	3.9 (3.5, 4.3)	4.0 (3.7, 4.2)
Intermediate	3.9 (3.4, 4.3)	4.7 (4.3, 5.1)	2.7 (2.4, 3.0)	3.4 (2.9, 3.9)	3.4 (2.9, 3.8)	3.7 (3.5, 3.9)
Tan	4.2 (3.6, 4.7)	4.3 (4.0, 4.7)	2.6 (2.3, 2.9)	3.2 (2.8, 3.7)	3.3 (2.8, 3.9)	3.6 (3.4, 3.8)
Brown	3.9 (3.4, 4.3)	4.4 (3.8, 5.0)	2.5 (2.3, 2.8)	4.2 (3.6, 4.9)	4.7 (3.6, 5.8)	4.0 (3.7, 4.4)
Dark	3.3 (2.9, 3.7)	5.1 (3.8, 6.5)	2.3 (2.1, 2.6)	4.6 (3.2, 5.9)	4.5 (2.4, 6.7)	4.1 (3.4, 4.8)
Overall	3.8 (3.5, 4.1)	4.7 (4.4, 5.0)	2.6 (2.4, 2.7)	4.0 (3.5, 4.5)	4.1 (3.4, 4.7)	3.9 (3.7, 4.1)
Difference between dark and light skin tones [3]	−0.1 (−0.5, 0.3)	−0.1 (−1.0, 0.8)	−0.6 (−1.0, −0.1)	0.6 (−0.5, 1.7)	0.6 (−0.8, 2.1)	0.2 (−0.3, 0.6)
Observed accuracy root mean square over $SaO_2 \leq 92\%$ (95% CI) [1]						
Very light or light	3.1 (2.2, 4.1)	5.3 (3.8, 6.8)	3.3 (1.2, 5.3)	4.5 (3.4, 5.7)	3.6 (2.5, 4.8)	4.4 (3.6, 5.2)
Intermediate	5.0 (1.8, 8.3)	4.2 (3.4, 4.9)	3.6 (1.9, 5.4)	4.6 (3.6, 5.5)	3.5 (2.3, 4.8)	4.2 (3.3, 5.1)
Tan	3.7 (2.9, 4.5)	4.0 (3.2, 4.8)	2.9 (2.0, 3.8)	5.2 (4.3, 6.1)	3.4 (2.4, 4.3)	3.9 (3.5, 4.4)
Brown	3.7 (3.1, 4.4)	5.8 (4.6, 7.0)	3.7 (2.9, 4.6)	5.4 (4.2, 6.6)	3.9 (2.9, 4.8)	4.7 (4.1, 5.4)
Dark	3.8 (2.2, 5.4)	3.5 (2.8, 4.2)	3.5 (2.9, 4.1)	6.8 (4.8, 8.7)	4.6 (1.5, 7.8)	4.6 (3.5, 5.7)
Overall	3.9 (3.2, 4.6)	4.9 (4.3, 5.5)	3.5 (2.9, 4.0)	5.4 (4.8, 6.0)	3.8 (3.1, 4.6)	4.4 (4.1, 4.8)
Adjusted accuracy root mean square over $SaO_2 \leq 92\%$ (95% CI) [2]						
Very light or light	3.5 (2.3, 4.7)	5.4 (4.5, 6.2)	3.7 (2.8, 4.5)	5.5 (4.1, 6.8)	4.5 (3.5, 5.5)	4.6 (4.0, 5.2)
Intermediate	4.2 (2.9, 5.5)	5.1 (4.3, 5.9)	3.7 (2.9, 4.6)	5.0 (3.9, 6.0)	4.1 (3.3, 5.0)	4.5 (3.8, 5.1)
Tan	4.7 (3.4, 6.1)	4.9 (4.2, 5.7)	3.7 (2.9, 4.5)	4.9 (4.0, 5.8)	4.2 (3.2, 5.1)	4.5 (3.9, 5.1)
Brown	4.2 (3.5, 5.0)	4.8 (3.9, 5.8)	3.5 (2.8, 4.2)	6.0 (5.2, 6.9)	5.7 (3.8, 7.6)	5.0 (4.4, 5.6)
Dark	3.9 (2.9, 4.9)	5.8 (4.5, 7.1)	3.4 (2.5, 4.3)	6.7 (5.4, 8.1)	5.7 (2.0, 9.4)	5.3 (4.1, 6.5)
Overall	4.2 (3.3, 5.1)	5.2 (4.6, 5.8)	3.6 (3.0, 4.2)	5.7 (5.1, 6.3)	5.0 (3.4, 6.6)	4.8 (4.4, 5.3)
Difference between dark and light skin tones [3]	0.4 (−0.8, 1.6)	0.5 (−1.1, 2.0)	−0.3 (−1.3, 0.8)	1.3 (−0.7, 3.2)	1.2 (−1.9, 4.4)	0.7 (−0.6, 2.0)

Pulse oximeters: A, ChoiceMMed Oxywatch MD300C19; B, ChoiceMMed Oxywatch MD300C13-CC12; C, Creative Medical PC-60B1-BL; D, Biolight Meditech M70C; E, Medlinket AM802 NHE.

[1] Root mean squared difference between SpO_2 and SaO_2 (accuracy root mean square; A_{RMS}).

[2] Square root of the expected squared difference between SpO_2 and SaO_2, where expected values were predicted using a fractional probit regression of the squared difference between SpO_2 and SaO_2 on SaO_2 and skin tone (individual typology angle, ITA), adjusting for pulse oximeter, haemoglobin and repeated observations within patients.

[3] Expected A_{RMS} for a patient with median dark skin tone (−44°) minus the expected A_{RMS} for a patient with median very light or light skin tone (46°), estimated using the model described above.

Supplementary table 5 False negative rate of two SpO_2 thresholds for predicting $SaO_2 \leq 92\%$, by skin tone

	Pulse oximeter					Overall
	A	B	C	D	E	
**False negative rate of $SpO_2 \leq 92\%$**						
Unadjusted, % (false negatives/cases) [1]						
Very light or light	11.1 (3/27)	12.7 (7/55)	30.0 (3/10)	53.7 (22/41)	8.7 (2/23)	23.7 (37/156)
Intermediate	36.4 (12/33)	15.2 (7/46)	27.8 (10/36)	58.6 (17/29)	30.3 (10/33)	31.6 (56/177)
Tan	32.1 (17/53)	13.5 (7/52)	37.8 (14/37)	78.0 (39/50)	21.6 (11/51)	36.2 (88/243)
Brown	39.0 (30/77)	25.0 (25/100)	28.8 (19/66)	67.1 (49/73)	31.6 (24/76)	37.5 (147/392)
Dark	35.0 (14/40)	26.7 (12/45)	58.3 (21/36)	70.0 (28/40)	39.5 (17/43)	45.1 (92/204)
Overall	33.0 (76/230)	19.5 (58/298)	36.2 (67/185)	66.5 (155/233)	28.3 (64/226)	35.8 (420/1172)
Adjusted, % (95% CI) [2]						
Very light or light	18.6 (11.5, 29.0)	4.6 (2.6, 7.9)	13.1 (7.4, 22.0)	56.1 (42.7, 69.3)	8.2 (4.6, 14.1)	20.1 (13.8, 28.5)
Intermediate	19.7 (13.6, 27.9)	5.8 (3.7, 9.1)	20.6 (14.0, 29.3)	67.0 (56.6, 76.4)	13.5 (8.9, 19.9)	25.3 (19.4, 32.5)
Tan	22.7 (15.5, 32.4)	8.5 (5.3, 13.5)	29.9 (20.7, 41.3)	76.9 (66.9, 85.0)	20.7 (13.9, 29.9)	31.7 (24.5, 40.4)
Brown	33.4 (23.7, 45.3)	18.4 (12.1, 27.3)	39.8 (28.4, 52.9)	85.2 (76.9, 91.3)	29.6 (20.5, 41.1)	41.3 (32.3, 51.6)
Dark	38.5 (26.3, 53.0)	17.8 (10.7, 28.3)	51.1 (36.8, 66.0)	83.0 (72.1, 90.7)	37.9 (24.7, 53.7)	45.7 (34.1, 58.4)
Overall	26.6 (18.1, 37.5)	11.0 (6.9, 17.2)	30.9 (21.5, 42.3)	73.6 (63.0, 82.5)	22.0 (14.5, 31.8)	32.8 (24.8, 42.3)
Difference between dark and light skin tones [3]	19.9 (7.1, 32.7)	13.2 (6.0, 20.4)	38.1 (24.6, 51.5)	26.8 (14.1, 39.6)	29.7 (14.7, 44.8)	25.5 (17.6, 33.5)
**False negative rate of $SpO_2 \leq 94\%$**						
Unadjusted, % (false negatives/cases) [1]						
Very light or light	0.0 (0/27)	5.5 (3/55)	0.0 (0/10)	26.8 (11/41)	0.0 (0/23)	9.0 (14/156)
Intermediate	15.2 (5/33)	6.5 (3/46)	5.6 (2/36)	27.6 (8/29)	6.1 (2/33)	11.3 (20/177)
Tan	13.2 (7/53)	1.9 (1/52)	16.2 (6/37)	48.0 (24/50)	7.8 (4/51)	17.3 (42/243)
Brown	18.2 (14/77)	13.0 (13/100)	13.6 (9/66)	42.5 (31/73)	15.8 (12/76)	20.2 (79/392)
Dark	15.0 (6/40)	11.1 (5/45)	27.8 (10/36)	55.0 (22/40)	7.0 (3/43)	22.5 (46/204)
Overall	13.9 (32/230)	8.4 (25/298)	14.6 (27/185)	41.2 (96/233)	9.3 (21/226)	17.2 (201/1172)
Adjusted, % (95% CI) [2]						
Very light or light	4.4 (2.4, 8.1)	1.6 (0.9, 3.1)	3.0 (1.5, 6.1)	26.9 (17.8, 39.0)	1.2 (0.6, 2.5)	7.4 (4.6, 11.8)
Intermediate	5.4 (3.4, 8.6)	2.2 (1.3, 3.7)	5.1 (3.0, 8.5)	32.6 (24.1, 42.7)	2.6 (1.5, 4.6)	9.5 (6.7, 13.6)
Tan	6.9 (4.3, 11.3)	3.3 (1.9, 5.8)	8.5 (5.1, 14.1)	39.0 (28.8, 50.7)	5.1 (2.9, 9.0)	12.6 (8.6, 18.2)
Brown	10.9 (6.8, 17.2)	7.0 (4.2, 11.6)	15.2 (9.5, 24.1)	47.9 (36.5, 60.1)	7.1 (4.2, 12.4)	17.6 (12.3, 25.1)
Dark	13.1 (7.7, 21.8)	6.9 (3.8, 12.7)	21.4 (12.9, 34.2)	62.2 (48.6, 74.9)	7.6 (4.0, 14.5)	22.2 (15.4, 31.6)
Overall	8.1 (4.9, 13.4)	4.2 (2.4, 7.4)	10.6 (6.4, 17.4)	41.7 (31.2, 53.5)	4.7 (2.6, 8.6)	13.9 (9.5, 20.1)
Difference between dark and light skin tones [3]	8.6 (3.5, 13.7)	5.3 (2.1, 8.5)	18.3 (8.7, 28.0)	35.3 (21.6, 49.1)	6.4 (2.4, 10.4)	14.8 (10.0, 19.6)

Pulse oximeters: A, ChoiceMMed Oxywatch MD300C19; B, ChoiceMMed Oxywatch MD300C13-CC12; C, Creative Medical PC-60B1-BL; D, Biolight Meditech M70C; E, Medlinket AM802 NHE.

[1] Proportion of cases of hypoxaemia ($SaO_2 \leq 92\%$) that test negative ($SpO_2 > 92\%$ or $SpO_2 > 94\%$; 'false negatives').

[2] Mean expected probability of testing negative ($SpO_2 > 92\%$ or $SpO_2 > 94\%$) among patients with hypoxaemia ($SaO_2 \leq 92\%$), estimated from a multi-level logistic regression of $SpO_2 \leq 92\%$ or $SpO_2 \leq 94\%$ on SaO_2 and skin tone (individual typology angle, ITA), adjusting for pulse oximeter and haemoglobin, with clustering of observations within patients within sites. Models fitted to all available observations, with expected probabilities averaged over observations of hypoxaemia ($SaO_2 \leq 92\%$) only.

[3] Expected false negative rate for a patient with median dark skin tone (−44°) minus the expected false negative rate for a patient with median very light or light skin tone (46°), estimated using the models described above.

Supplementary table 6 False positive rate of two SpO_2 thresholds for predicting $SaO_2 \leq 92\%$, by skin tone

	Pulse oximeter					Overall
	A	B	C	D	E	
**False positive rate of $SpO_2 \leq 92\%$**						
Unadjusted, % (false positives/non-cases) [1]						
Very light or light	16.4 (35/214)	40.6 (116/286)	14.2 (22/155)	9.2 (26/284)	21.6 (47/218)	21.3 (246/1157)
Intermediate	16.0 (52/326)	36.7 (101/275)	9.7 (29/299)	4.9 (14/283)	18.8 (61/325)	17.0 (257/1508)
Tan	15.9 (65/410)	31.6 (130/412)	8.4 (34/405)	3.4 (15/446)	17.9 (82/459)	15.3 (326/2132)
Brown	15.1 (101/667)	22.8 (153/670)	7.5 (45/601)	6.0 (47/786)	13.9 (105/756)	13.0 (451/3480)
Dark	10.6 (35/330)	21.5 (59/274)	6.3 (23/365)	2.5 (8/323)	10.9 (29/267)	9.9 (154/1559)
Overall	14.8 (288/1947)	29.2 (559/1917)	8.4 (153/1825)	5.2 (110/2122)	16.0 (324/2025)	14.6 (1434/9836)
Adjusted, % (95% CI) [2]						
Very light or light	21.3 (14.8, 29.6)	45.0 (35.3, 55.5)	17.1 (11.2, 25.0)	6.0 (3.5, 10.2)	29.6 (21.9, 38.8)	23.8 (17.3, 31.8)
Intermediate	22.7 (17.2, 29.4)	43.5 (35.4, 52.3)	12.7 (9.0, 17.6)	4.4 (2.8, 7.1)	23.5 (18.1, 29.9)	21.4 (16.5, 27.2)
Tan	22.9 (17.0, 30.1)	39.0 (30.8, 48.2)	9.6 (6.4, 14.0)	3.3 (2.0, 5.6)	18.8 (13.9, 24.7)	18.7 (14.0, 24.5)
Brown	18.9 (13.6, 25.7)	26.7 (19.8, 34.9)	7.9 (5.0, 12.1)	2.6 (1.6, 4.4)	15.9 (11.4, 21.7)	14.4 (10.3, 19.8)
Dark	14.7 (9.7, 21.4)	25.0 (17.3, 34.6)	4.7 (2.7, 8.0)	2.8 (1.5, 5.3)	11.0 (6.7, 17.2)	11.6 (7.6, 17.3)
Overall	20.1 (14.5, 27.2)	35.8 (27.7, 45.1)	10.4 (6.9, 15.3)	3.8 (2.3, 6.5)	19.8 (14.4, 26.5)	18.0 (13.1, 24.1)
Difference between dark and light skin tones [3]	−6.7 (−12.7, −0.7)	−19.9 (−28.3, −11.6)	−12.4 (−17.4, −7.3)	−3.2 (−5.4, −1.0)	−18.7 (−25.8, −11.5)	−12.2 (−14.9, −9.5)
**False positive rate of $SpO_2 \leq 94\%$**						
Unadjusted, % (false positives/non-cases) [1]						
Very light or light	25.2 (54/214)	47.6 (136/286)	25.2 (39/155)	10.9 (31/284)	36.2 (79/218)	29.3 (339/1157)
Intermediate	29.8 (97/326)	40.7 (112/275)	18.1 (54/299)	7.4 (21/283)	26.8 (87/325)	24.6 (371/1508)
Tan	28.0 (115/410)	43.7 (180/412)	13.1 (53/405)	8.1 (36/446)	27.2 (125/459)	23.9 (509/2132)
Brown	24.0 (160/667)	27.9 (187/670)	11.1 (67/601)	7.3 (57/786)	20.2 (153/756)	17.9 (624/3480)
Dark	16.4 (54/330)	29.6 (81/274)	7.1 (26/365)	2.5 (8/323)	12.4 (33/267)	13.0 (202/1559)
Overall	24.7 (480/1947)	36.3 (696/1917)	13.1 (239/1825)	7.2 (153/2122)	23.6 (477/2025)	20.8 (2045/9836)
Adjusted, % (95% CI) [2]						
Very light or light	51.3 (41.8, 61.0)	73.1 (64.3, 80.7)	43.7 (34.5, 53.7)	16.5 (11.3, 23.4)	65.1 (56.7, 73.2)	49.9 (41.7, 58.4)
Intermediate	51.7 (44.3, 59.3)	72.1 (65.0, 78.5)	38.3 (31.8, 45.3)	15.6 (11.6, 20.8)	56.1 (49.8, 62.5)	46.8 (40.5, 53.3)
Tan	50.7 (42.7, 58.8)	68.7 (60.7, 76.0)	32.6 (26.3, 39.7)	14.8 (10.6, 20.3)	48.7 (42.2, 55.5)	43.1 (36.5, 50.0)
Brown	44.2 (36.3, 52.5)	56.7 (48.1, 65.1)	25.4 (19.6, 32.1)	13.0 (9.2, 18.0)	46.7 (40.1, 53.7)	37.2 (30.7, 44.3)
Dark	35.3 (27.3, 44.1)	50.9 (40.8, 61.2)	17.8 (12.8, 23.9)	6.8 (4.2, 10.9)	41.6 (33.6, 50.0)	30.5 (23.7, 38.0)
Overall	46.7 (38.5, 55.1)	64.3 (55.8, 72.3)	31.5 (25.0, 38.9)	13.3 (9.4, 18.7)	51.7 (44.5, 59.0)	41.5 (34.6, 48.8)
Difference between dark and light skin tones [3]	−16.1 (−24.6, −7.6)	−22.1 (−31.5, −12.8)	−25.9 (−34.0, −17.9)	−9.7 (−14.6, −4.8)	−23.5 (−31.9, −15.2)	−19.5 (−23.0, −15.9)

Pulse oximeters: A, ChoiceMMed Oxywatch MD300C19; B, ChoiceMMed Oxywatch MD300C13-CC12; C, Creative Medical PC-60B1-BL; D, Biolight Meditech M70C; E, Medlinket AM802 NHE.

[1] Proportion of non-cases of hypoxaemia ($SaO_2 > 92\%$) that test positive ($SpO_2 \leq 92\%$ or $SpO_2 \leq 94\%$; 'false positives').

[2] Mean expected probability of testing positive ($SpO_2 \leq 92\%$ or $SpO_2 \leq 94\%$) among patients without hypoxaemia ($SaO_2 > 92\%$), estimated from a multi-level logistic regression of $SpO_2 \leq 92\%$ or $SpO_2 \leq 94\%$ on SaO_2 and skin tone (individual typology angle, ITA), adjusting for pulse oximeter and haemoglobin, with clustering of observations within patients within sites (i.e. the same models as for false negative rate). Models fitted to all available observations, with expected probabilities averaged over observations without hypoxaemia ($SaO_2 > 92\%$) only.

[3] Expected false positive rate for a patient with median dark skin tone (−44°) minus the expected false positive rate for a patient with median very light or light skin tone (46°), estimated using the models described above.

Supplementary table 7 Area under the receiver operating characteristic curve of SpO_2 for predicting $SaO_2 \leq 92\%$, by skin tone

	Pulse oximeter					Overall
	A	B	C	D	E	
Unadjusted area under the ROC curve (95% CI) [1]						
Very light or light	0.87 (0.80, 0.93)	0.76 (0.70, 0.82)	0.81 (0.70, 0.92)	0.70 (0.63, 0.78)	0.86 (0.80, 0.92)	0.77 (0.73, 0.81)
Intermediate	0.73 (0.65, 0.81)	0.82 (0.75, 0.88)	0.84 (0.79, 0.89)	0.74 (0.61, 0.87)	0.83 (0.77, 0.89)	0.79 (0.74, 0.83)
Tan	0.77 (0.70, 0.84)	0.86 (0.81, 0.90)	0.86 (0.80, 0.91)	0.78 (0.70, 0.85)	0.80 (0.74, 0.86)	0.79 (0.75, 0.83)
Brown	0.74 (0.67, 0.81)	0.76 (0.72, 0.80)	0.89 (0.84, 0.94)	0.72 (0.65, 0.78)	0.80 (0.74, 0.85)	0.77 (0.74, 0.80)
Dark	0.82 (0.75, 0.88)	0.77 (0.67, 0.87)	0.88 (0.82, 0.93)	0.68 (0.60, 0.75)	0.74 (0.59, 0.90)	0.76 (0.71, 0.81)
Overall	0.77 (0.73, 0.80)	0.78 (0.75, 0.81)	0.87 (0.84, 0.89)	0.72 (0.68, 0.75)	0.80 (0.76, 0.84)	0.77 (0.75, 0.79)
Adjusted area under the ROC curve (95% CI) [2]						
Very light or light	0.83 (0.78, 0.87)	0.90 (0.87, 0.93)	0.79 (0.73, 0.85)	0.66 (0.60, 0.72)	0.86 (0.82, 0.90)	0.81 (0.78, 0.84)
Intermediate	0.81 (0.77, 0.85)	0.90 (0.87, 0.92)	0.77 (0.73, 0.82)	0.66 (0.60, 0.71)	0.85 (0.82, 0.88)	0.80 (0.78, 0.83)
Tan	0.78 (0.73, 0.83)	0.89 (0.86, 0.92)	0.77 (0.72, 0.81)	0.63 (0.57, 0.69)	0.84 (0.80, 0.87)	0.78 (0.76, 0.81)
Brown	0.74 (0.68, 0.80)	0.85 (0.81, 0.90)	0.79 (0.73, 0.85)	0.53 (0.44, 0.63)	0.79 (0.72, 0.85)	0.75 (0.70, 0.79)
Dark	0.76 (0.70, 0.81)	0.82 (0.77, 0.88)	0.74 (0.66, 0.82)	0.65 (0.46, 0.83)	0.82 (0.75, 0.88)	0.75 (0.70, 0.81)
Overall	0.77 (0.74, 0.80)	0.87 (0.85, 0.89)	0.77 (0.74, 0.81)	0.62 (0.58, 0.66)	0.82 (0.79, 0.85)	0.77 (0.76, 0.79)
Difference between dark and light skin tones [3]	−0.07 (−0.13, −0.01)	−0.07 (−0.13, −0.02)	−0.05 (−0.14, 0.04)	−0.01 (−0.15, 0.13)	−0.04 (−0.10, 0.02)	−0.06 (−0.10, −0.02)

Pulse oximeters: A, ChoiceMMed Oxywatch MD300C19; B, ChoiceMMed Oxywatch MD300C13-CC12; C, Creative Medical PC-60B1-BL; D, Biolight Meditech M70C; E, Medlinket AM802 NHE.

[1] Estimated using nonparametric analysis of receiver operating characteristic (ROC) curve with bootstrapped confidence intervals, among patients with the specified combination of skin tone category and oximeter only.

[2] Estimated from a probit ROC regression model, with fixed effects of pulse oximeter model and ITA, fixed effect interactions between pulse oximeter model, skin tone (individual typology angle, ITA), with control distributions adjusted for haemoglobin and the variance-covariance matrix clustered at the patient level. Overall estimates obtained from separate models excluding the fixed effects and interactions for pulse oximeter model, ITA, or both.

[3] Expected area under the ROC curve for a patient with median dark skin tone (−44°) minus the expected area under the ROC curve for a patient with median very light or light skin tone (46°), estimated using the models described above.

Supplementary table 8 Occult hypoxaemia by skin tone

	Pulse oximeter					Overall
	A	B	C	D	E	
Prevalence of $SpO_2 > 92\%$ among patients with $SaO_2 < 88\%$						
Unadjusted, % (false negatives/cases) [1]						
Very light or light	0.0 (0/2)	0.0 (0/5)	0.0 (0/2)	0.0 (0/5)	0.0 (0/3)	0.0 (0/17)
Intermediate	25.0 (1/4)	0.0 (0/2)	0.0 (0/6)	25.0 (1/4)	0.0 (0/5)	9.5 (2/21)
Tan	14.3 (1/7)	0.0 (0/8)	33.3 (1/3)	42.9 (3/7)	9.1 (1/11)	16.7 (6/36)
Brown	0.0 (0/12)	6.2 (1/16)	14.3 (2/14)	23.1 (3/13)	6.2 (1/16)	9.9 (7/71)
Dark	20.0 (1/5)	0.0 (0/5)	33.3 (1/3)	16.7 (1/6)	22.2 (2/9)	17.9 (5/28)
Overall	10.0 (3/30)	2.8 (1/36)	14.3 (4/28)	22.9 (8/35)	9.1 (4/44)	11.6 (20/173)
Adjusted, % (95% CI) [2]						
Very light or light	2.3 (0.7, 7.3)	0.2 (0.1, 0.9)	0.6 (0.2, 2.4)	15.3 (6.2, 33.4)	0.6 (0.2, 2.2)	3.8 (1.5, 9.2)
Intermediate	3.1 (1.2, 8.0)	0.4 (0.1, 1.2)	1.4 (0.5, 4.4)	26.8 (14.5, 44.8)	1.4 (0.5, 4.0)	6.6 (3.4, 12.5)
Tan	4.9 (2.0, 12.1)	0.7 (0.2, 2.5)	3.2 (1.1, 9.2)	43.8 (26.8, 63.3)	3.2 (1.2, 8.7)	11.2 (6.2, 19.2)
Brown	11.9 (5.2, 25.7)	2.8 (0.9, 8.2)	7.2 (2.6, 18.9)	65.7 (45.8, 82.7)	7.6 (3.0, 18.4)	19.0 (11.5, 30.8)
Dark	14.7 (5.8, 33.5)	2.7 (0.8, 8.9)	11.6 (3.9, 30.4)	62.3 (39.8, 82.3)	11.0 (3.9, 27.9)	20.4 (10.8, 36.6)
Overall	7.4 (3.0, 17.3)	1.4 (0.4, 4.3)	4.8 (1.6, 13.1)	42.8 (26.6, 61.3)	4.8 (1.8, 12.3)	12.2 (6.7, 21.6)
Difference between dark and light skin tones [3]	12.4 (−1.3, 26.1)	2.5 (−0.9, 5.9)	10.9 (−2.3, 24.2)	47.0 (18.3, 75.7)	10.4 (−3.9, 24.6)	16.6 (5.6, 27.7)

Pulse oximeters: A, ChoiceMMed Oxywatch MD300C19; B, ChoiceMMed Oxywatch MD300C13-CC12; C, Creative Medical PC-60B1-BL; D, Biolight Meditech M70C; E, Medlinket AM802 NHE.

[1] Proportion of cases of severe hypoxaemia ($SaO_2 < 88\%$) that test negative ($SpO_2 > 92\%$; 'false negatives').

[2] Mean expected probability of testing negative ($SpO_2 > 92\%$) among patients with severe hypoxaemia ($SaO_2 < 88\%$), estimated from a multi-level logistic regression of $SpO_2 \leq 92\%$ on SaO_2 and skin tone (individual typology angle, ITA), adjusting for pulse oximeter and haemoglobin, with clustering of observations within patients within sites (i.e. the same model as for false negative rate of $SpO_2 \leq 92\%$). Model fitted to all available observations, with expected probabilities averaged over observations of severe hypoxaemia (SaO_2 <88%) only.

[3] Expected rate of occult hypoxaemia for a patient with median dark skin tone (−44°) minus the expected rate of occult hypoxaemia for a patient with median very light or light skin tone (46°), estimated using the model described above.

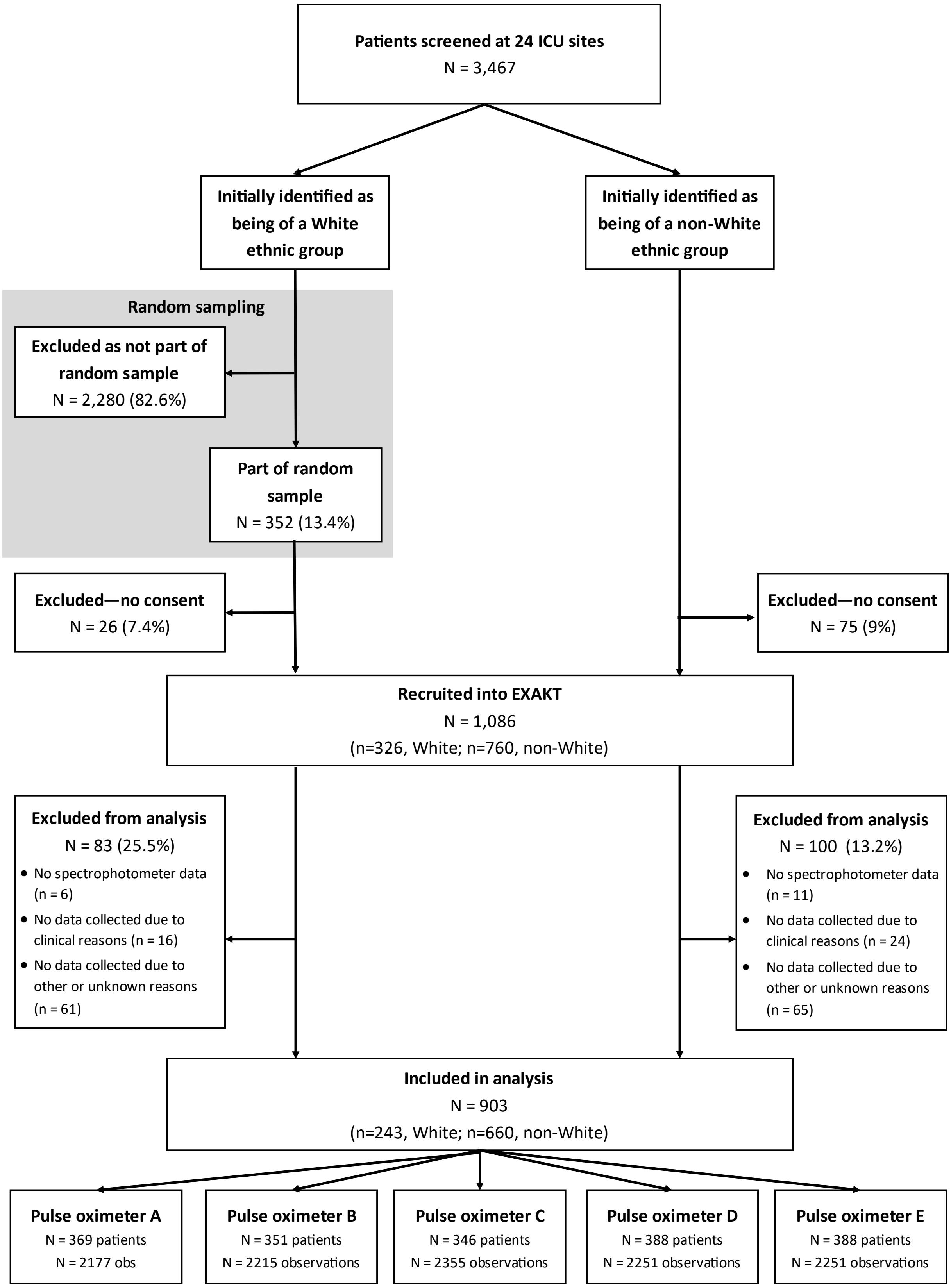

Supplementary figure 1 Recruitment flow diagram

Pulse oximeters: A, ChoiceMMed Oxywatch MD300C19; B, ChoiceMMed Oxywatch MD300C13-CC12; C, Creative Medical PC-60B1-BL; D, Biolight Meditech M70C; E, Medlinket AM802 NHE. ICU: intensive care unit.
Initial identification of White/non-White ethnic group was conducted for screening purposes only and may differ from the ethnic groups identified in Table 1, which were ultimately derived from electronic health records. Each patient was randomly assigned up to two pulse oximeters.

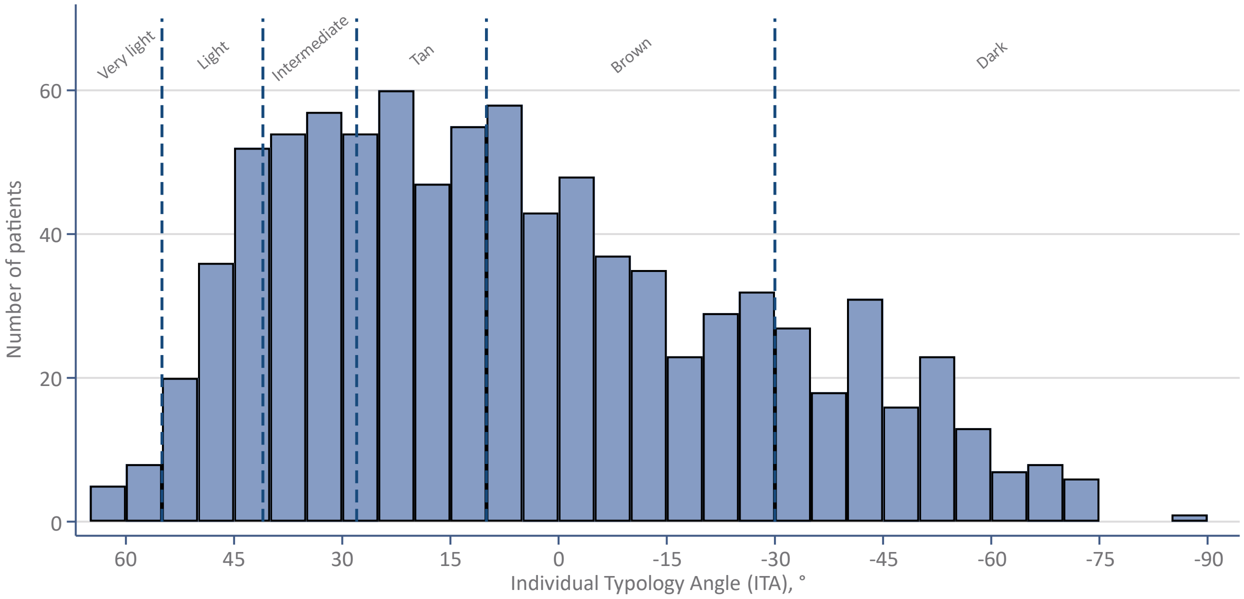

Supplementary figure 2 Distribution of individual typology angle (ITA) in the EXAKT study cohort

Each bar represents the number of patients with an individual typology angle (ITA) within the 5° range indicated by the width of the bar. Vertical dashed lines represent the previously defined categories of skin tone based on ITA used in descriptive results.

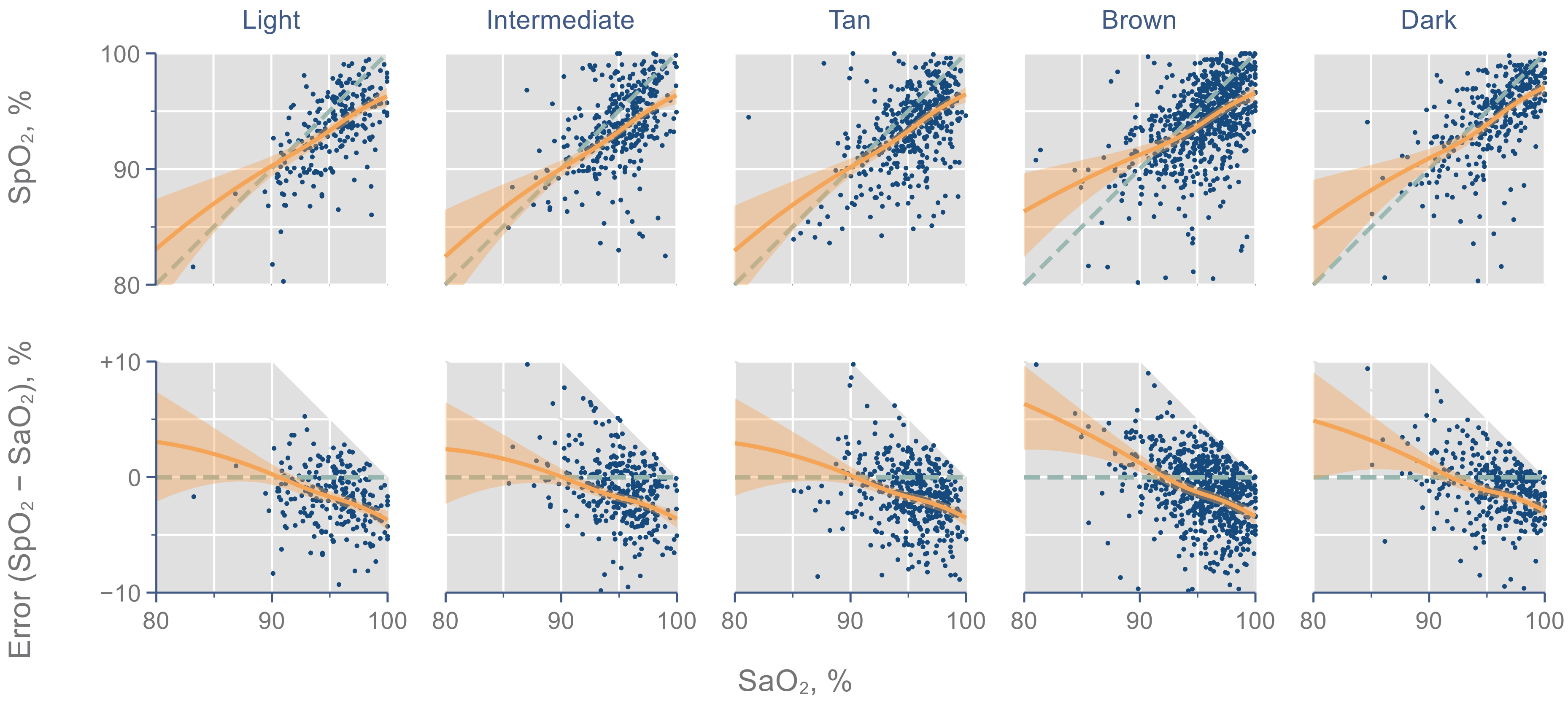

Supplementary figure 3A Relationship between SpO_2 and SaO_2, by skin tone category (pulse oximeter A)

Each dot represents one observation, illustrating the relationship between SpO_2 and SaO_2, across the range of SaO_2 from 80% to 100%, with the dashed line indicating exact agreement. In the top row, the data are presented with SpO_2 on the vertical axis, the same data are presented with the difference between SpO_2 and SaO_2 (error or bias) on the vertical axis. Solid lines illustrate the adjusted mean error (bias) with shaded 95% confidence interval. Pulse oximeter A, ChoiceMMed Oxywatch MD300C19.

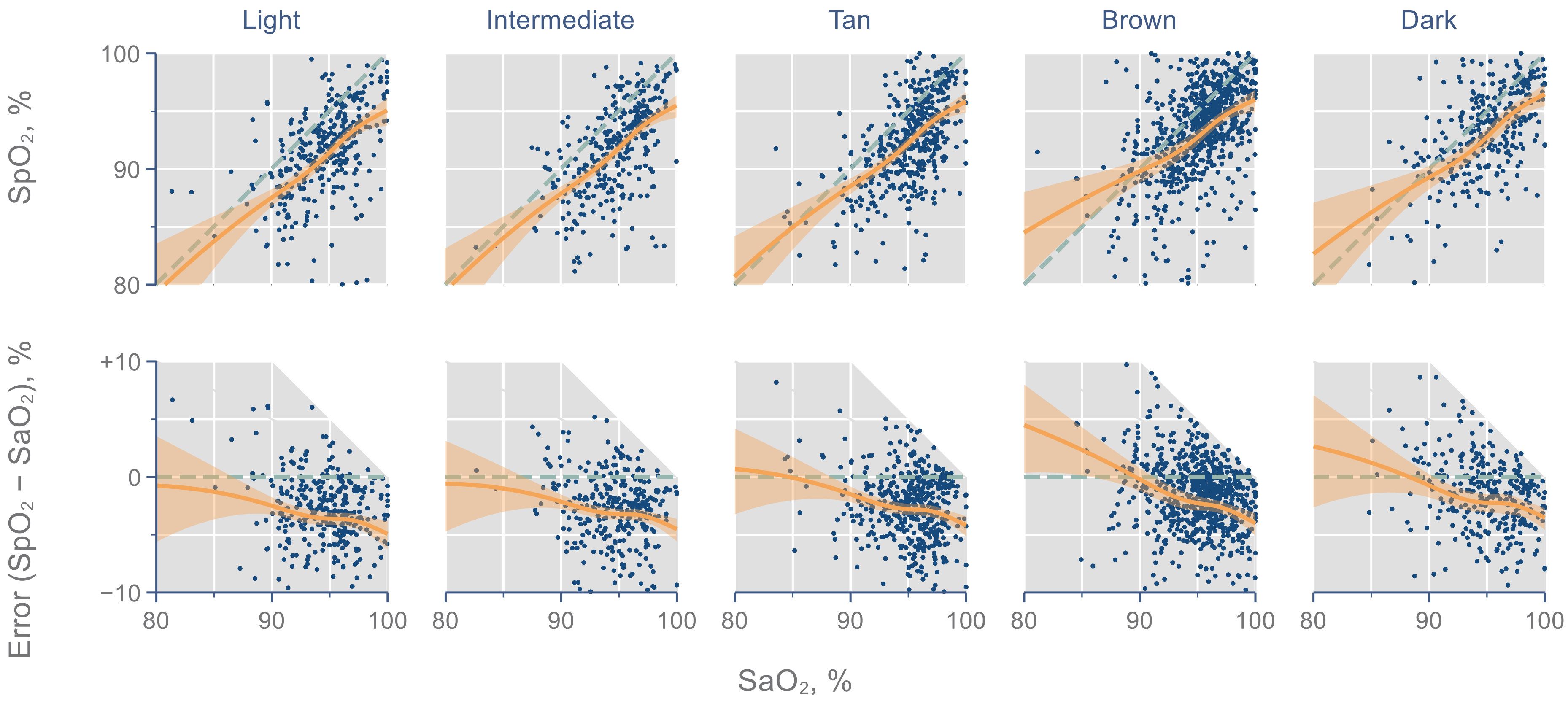

Supplementary figure 3B Relationship between SpO_2 and SaO_2, by skin tone category (pulse oximeter B)

Each dot represents one observation, illustrating the relationship between SpO_2 and SaO_2, across the range of SaO_2 from 80% to 100%, with the dashed line indicating exact agreement. In the top row, the data are presented with SpO_2 on the vertical axis, the same data are presented with the difference between SpO_2 and SaO_2 (error or bias) on the vertical axis. Solid lines illustrate the adjusted mean error (bias) with shaded 95% confidence intervals. Pulse oximeter B, ChoiceMMed Oxywatch MD300C13-CC12.

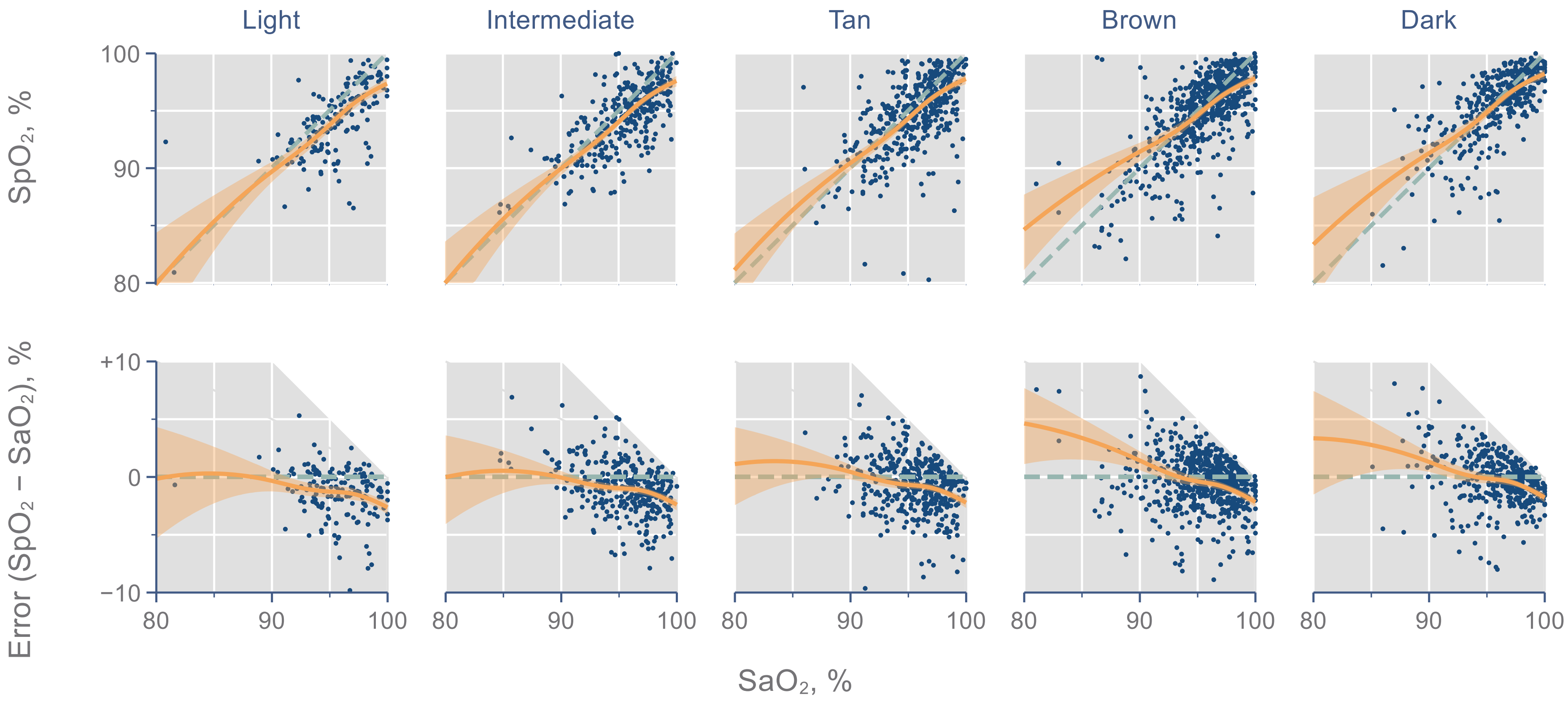

Supplementary figure 3C Relationship between SpO_2 and SaO_2, by skin tone category (pulse oximeter C)

Each dot represents one observation, illustrating the relationship between SpO_2 and SaO_2, across the range of SaO_2 from 80% to 100%, with the dashed line indicating exact agreement. In the top row, the data are presented with SpO_2 on the vertical axis, the same data are presented with the difference between SpO_2 and SaO_2 (error or bias) on the vertical axis. Solid lines illustrate the adjusted mean error (bias) with shaded 95% confidence intervals. Pulse oximeter C, Creative Medical PC-60B1-BL.

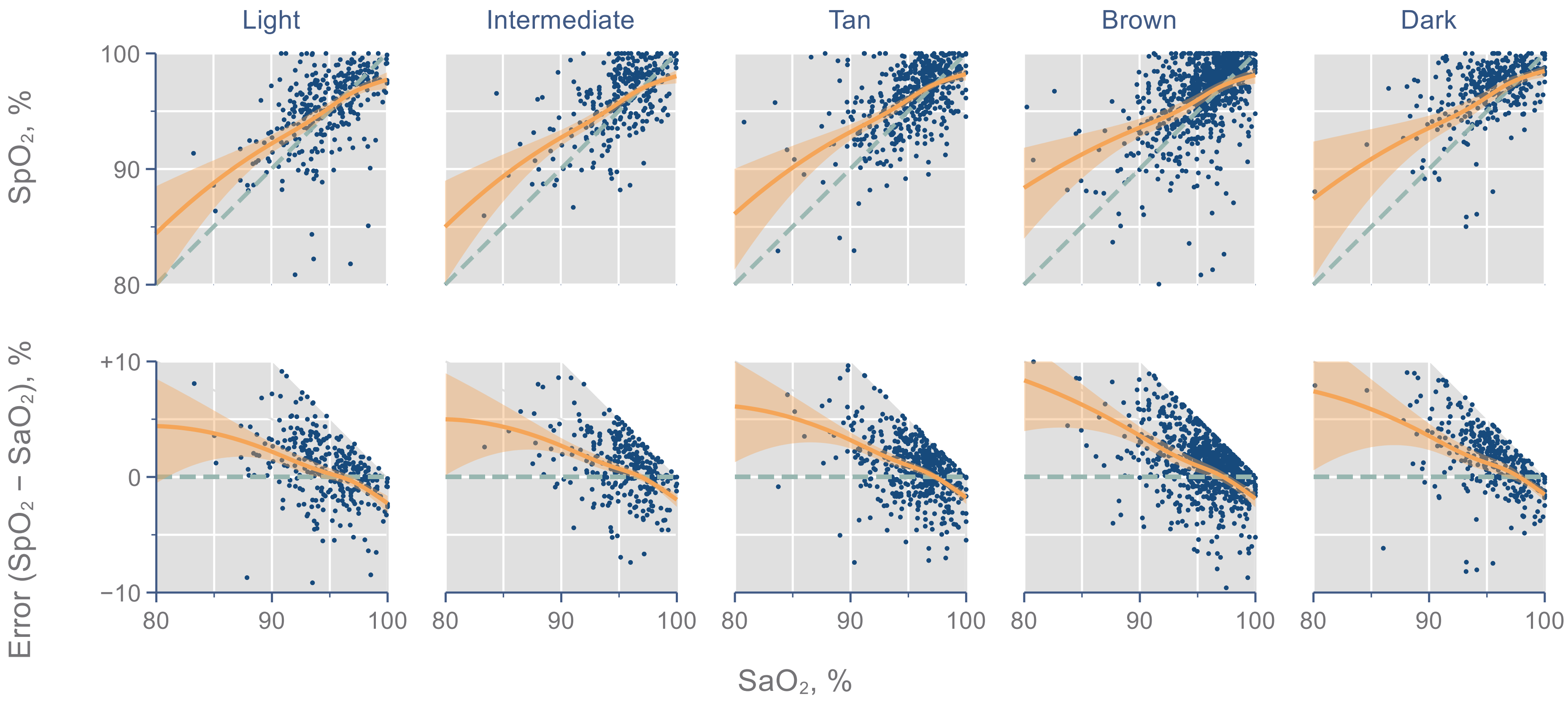

Supplementary figure 3D Relationship between SpO_2 and SaO_2, by skin tone category (pulse oximeter D)

Each dot represents one observation, illustrating the relationship between SpO_2 and SaO_2, across the range of SaO_2 from 80% to 100%, with the dashed line indicating exact agreement. In the top row, the data are presented with SpO_2 on the vertical axis, the same data are presented with the difference between SpO_2 and SaO_2 (error or bias) on the vertical axis. Solid lines illustrate the adjusted mean error (bias) with shaded 95% confidence intervals. Pulse oximeter D, Biolight Meditech M70C.

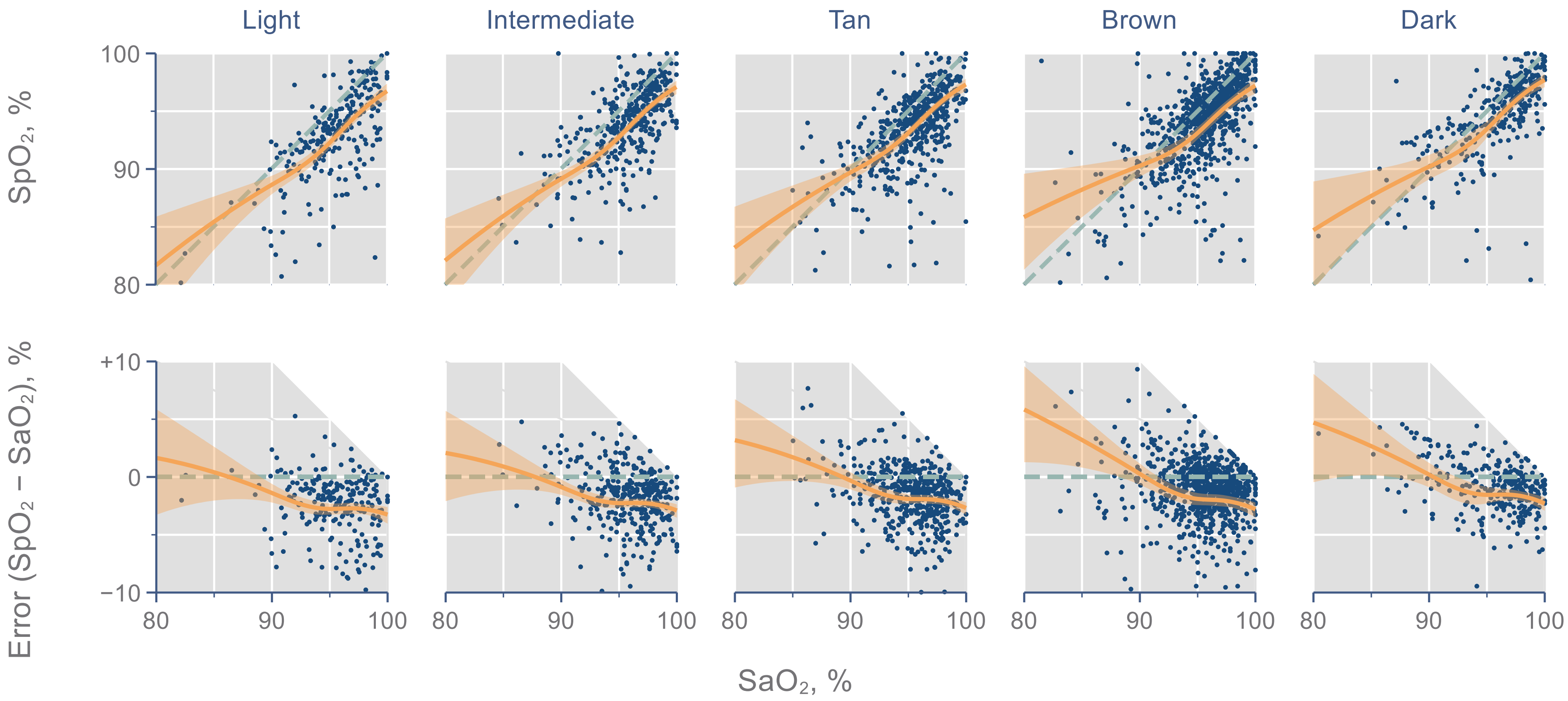

Supplementary figure 3E Relationship between SpO_2 and SaO_2, by skin tone category (pulse oximeter E)

Each dot represents one observation, illustrating the relationship between SpO_2 and SaO_2, across the range of SaO_2 from 80% to 100%, with the dashed line indicating exact agreement. In the top row, the data are presented with SpO_2 on the vertical axis, the same data are presented with the difference between SpO_2 and SaO_2 (error or bias) on the vertical axis. Solid lines illustrate the adjusted mean error (bias) with shaded 95% confidence intervals. Pulse oximeter E, Medlinket AM802 NHE.

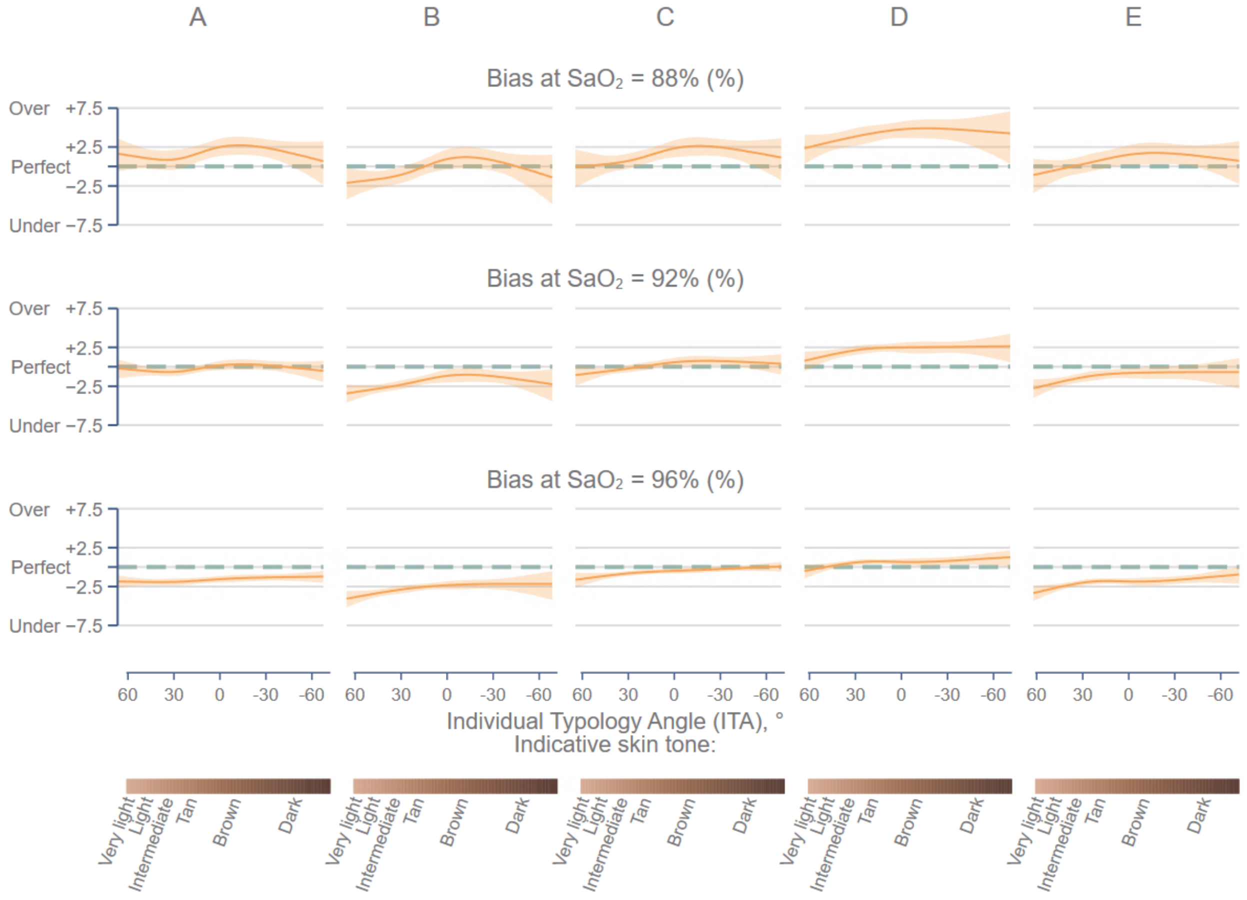

Supplementary figure 4 Relationship between bias and skin tone at different levels of SaO_2

Solid lines indicate modelled estimates, with shading of 95% confidence limits. Dashed lines indicate exact agreement or perfect prediction. See Detailed methods for statistical analysis for model details and estimates at different levels of SaO_2 and SpO_2. Pulse oximeters: A, ChoiceMMed Oxywatch MD300C19; B, ChoiceMMed Oxywatch MD300C13-CC12; C, Creative Medical PC-60B1-BL; D, Biolight Meditech M70C; E, Medlinket AM802 NHE.

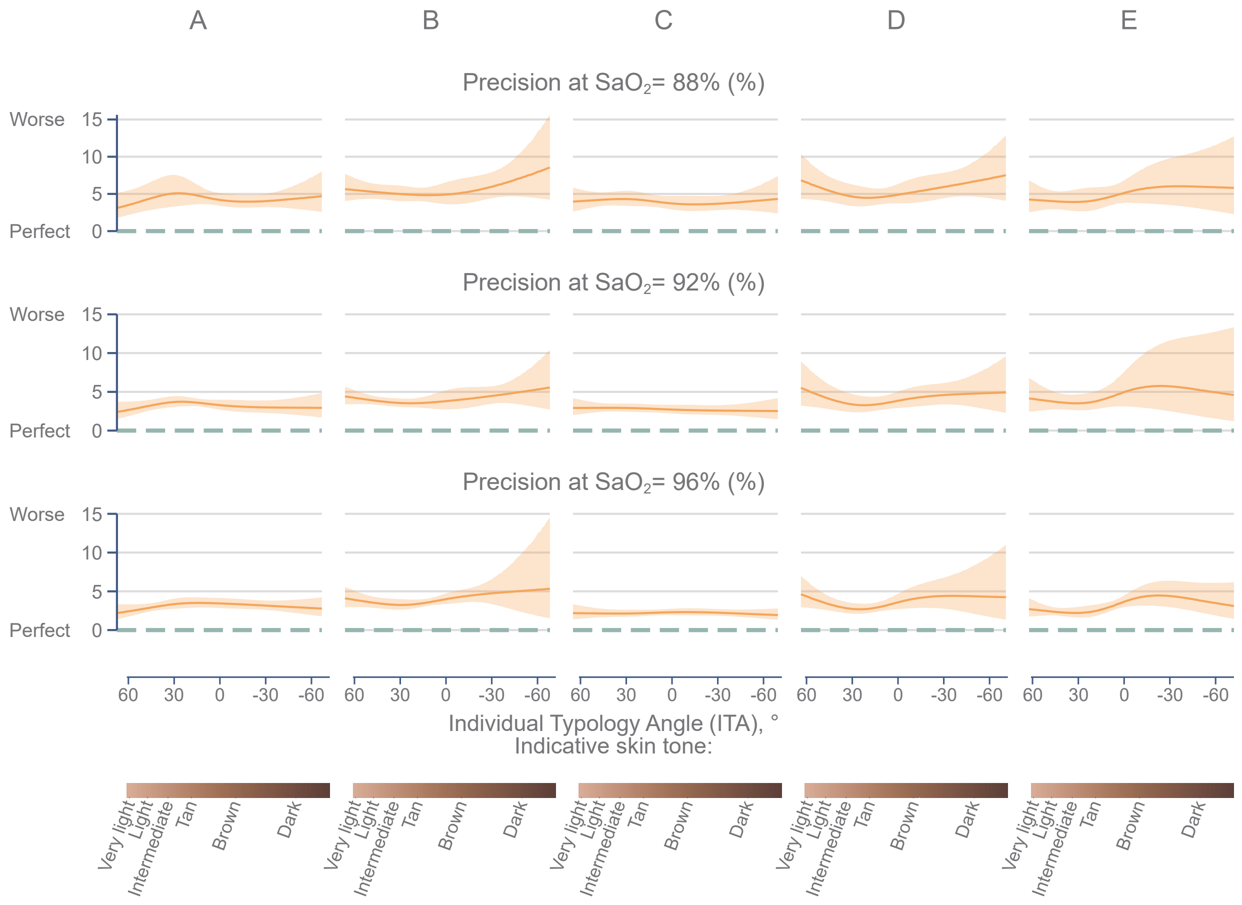

Supplementary figure 5 Relationship between precision and skin tone at different levels of SaO_2

Solid lines indicate modelled estimates, with shading of 95% confidence limits. Dashed lines indicate exact agreement or perfect prediction. See Detailed methods for statistical analysis for model details and estimates at different levels of SaO_2 and SpO_2. Pulse oximeters: A, ChoiceMMed Oxywatch MD300C19; B, ChoiceMMed Oxywatch MD300C13-CC12; C, Creative Medical PC-60B1-BL; D, Biolight Meditech M70C; E, Medlinket AM802 NHE.

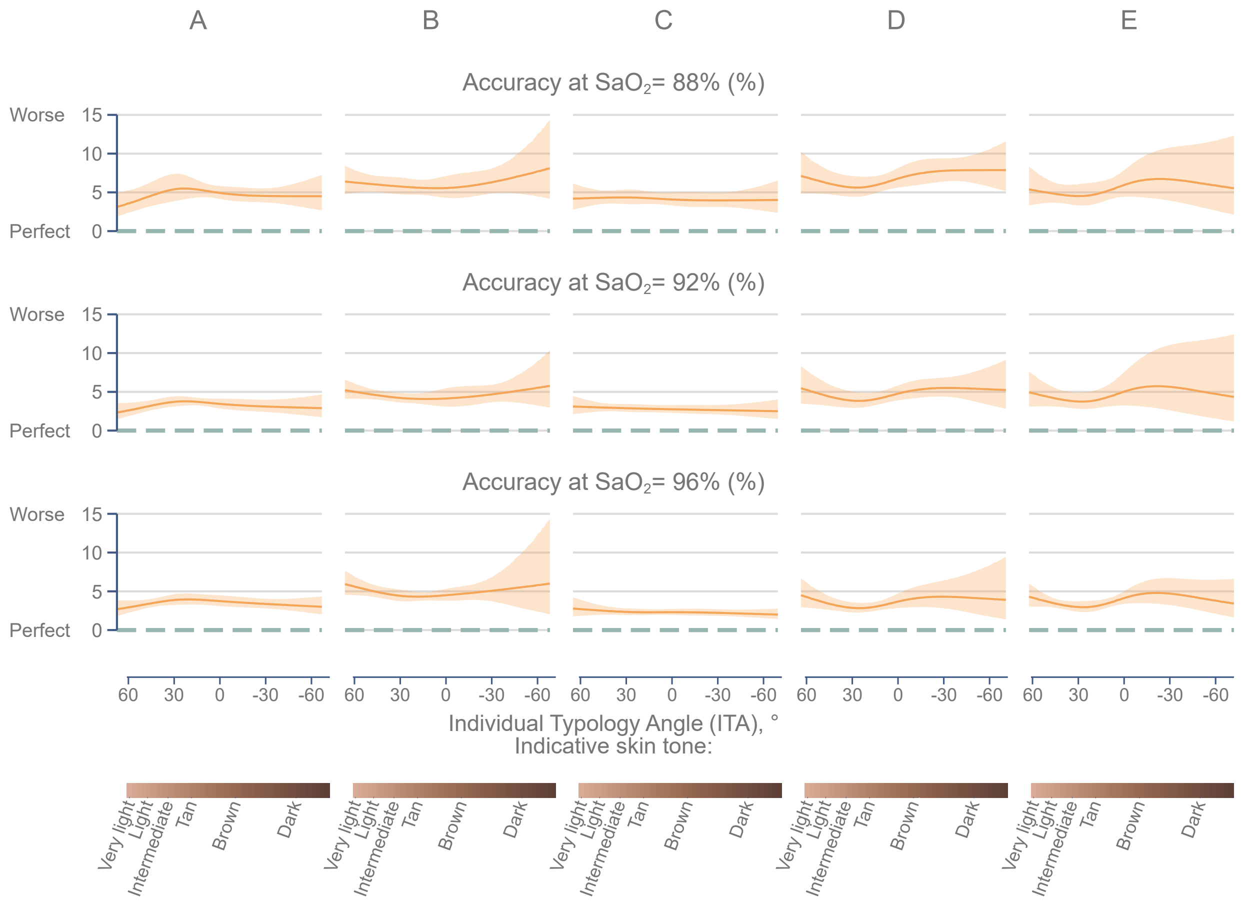

Supplementary figure 6 Relationship between accuracy and skin tone at different levels of SaO_2

Solid lines indicate modelled estimates, with shading of 95% confidence limits. Dashed lines indicate exact agreement or perfect prediction. See Detailed methods for statistical analysis for model details and estimates at different levels of SaO_2 and SpO_2. Pulse oximeters: A, ChoiceMMed Oxywatch MD300C19; B, ChoiceMMed Oxywatch MD300C13-CC12; C, Creative Medical PC-60B1-BL; D, Biolight Meditech M70C; E, Medlinket AM802 NHE.

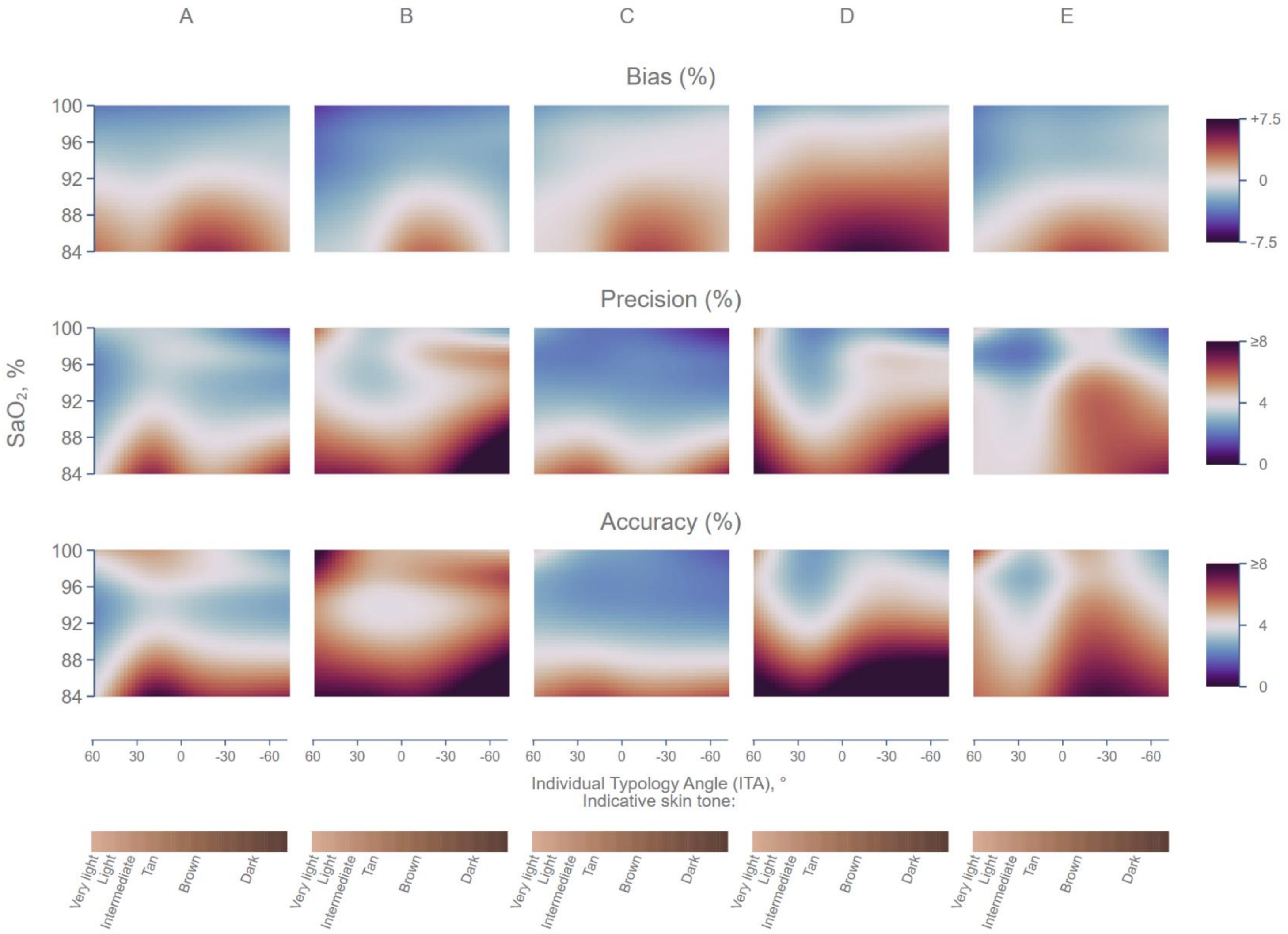

Supplementary figure 7 Heat maps showing variation in bias, precision and accuracy across combinations of skin tone and SaO_2

Bias: mean error (systematic error); Precision: root mean squared deviation (random error). Accuracy: accuracy root mean square (total error). For bias, white shading indicates the optimum (0%), with red shading indicating positive bias and blue shading negative bias. For precision and accuracy, white indicates the recommended upper limit for accuracy (4%), red shading indicates values exceeding this limit and blue shading values within this limit (with darkest blue indicating the optimum of 0%). Note that heatmaps illustrate the point estimates only and do not illustrate uncertainty (confidence intervals) surrounding the curves. Pulse oximeters: A, ChoiceMMed Oxywatch MD300C19; B, ChoiceMMed Oxywatch MD300C13-CC12; C, Creative Medical PC-60B1-BL; D, Biolight Meditech M70C; E, Medlinket AM802 NHE.

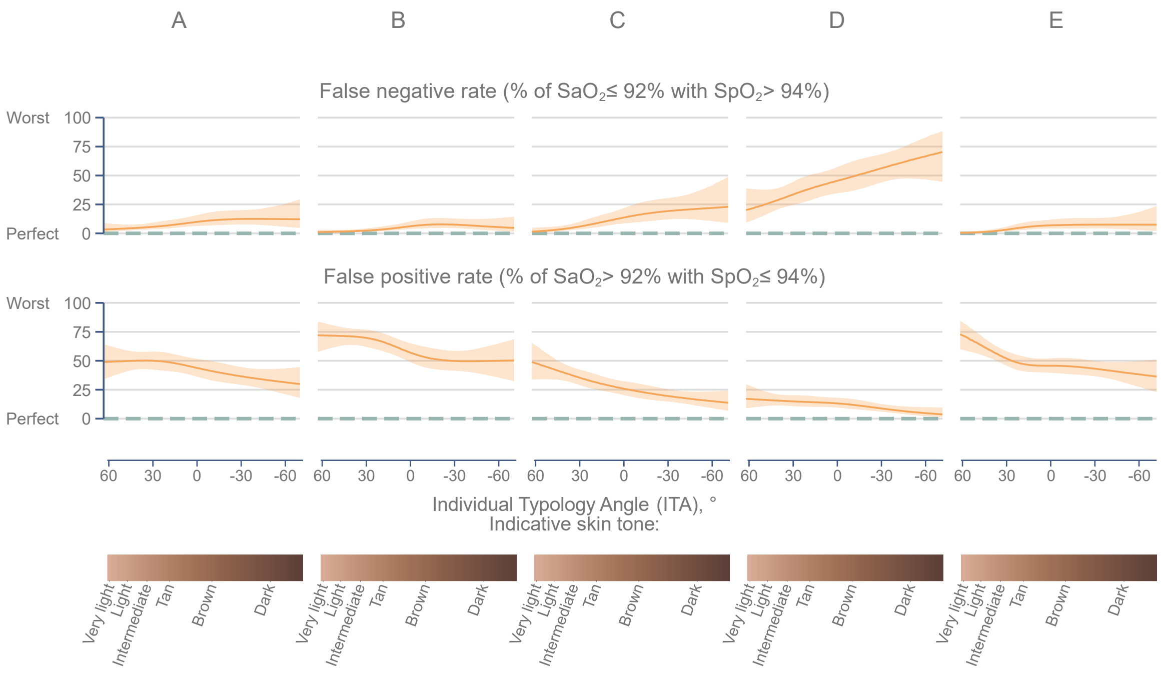

Supplementary figure 8 False negative rate and false positive rate of $SpO_2 \leq 94\%$ for predicting $SaO_2 \leq 92\%$

Solid lines indicate modelled estimates, with shading of 95% confidence limits. Dashed lines indicate exact agreement or perfect prediction. See Detailed methods for statistical analysis for model details and estimates at different levels of SaO_2 and SpO_2. Pulse oximeters: A, ChoiceMMed Oxywatch MD300C19; B, ChoiceMMed Oxywatch MD300C13-CC12; C, Creative Medical PC-60B1-BL; D, Biolight Meditech M70C; E, Medlinket AM802 NHE.
